# Investigating the nature of prokaryotic genomic island locations within a genome

**DOI:** 10.1371/journal.pone.0301172

**Published:** 2024-05-02

**Authors:** Reem Aldaihani, Lenwood S. Heath

**Affiliations:** 1 Department of Computer Science, Kuwait University, Kuwait City, State of Kuwait, Kuwait; 2 Department of Computer Science, Virginia Tech, Blacksburg, VA, United States of America; Gandhi Insititute of Technology and Management, INDIA

## Abstract

Horizontal gene transfer (HGT) is a powerful evolutionary force that considerably shapes the structure of prokaryotic genomes and is associated with genomic islands (GIs). A GI is a DNA segment composed of transferred genes that can be found within a prokaryotic genome, obtained through HGT. Much research has focused on detecting GIs in genomes, but here we pursue a new course, which is identifying possible preferred locations of GIs in the prokaryotic genome. Here, we identify the locations of the GIs within prokaryotic genomes to examine patterns in those locations. Prokaryotic GIs were analyzed according to the genome structure that they are located in, whether it be a circular or a linear genome. The analytical investigations employed are: (1) studying the GI locations in relation to the origin of replication (oriC); (2) exploring the distances between GIs; and (3) determining the distribution of GIs across the genomes. For each of the investigations, the analysis was performed on all of the GIs in the data set. Moreover, to void bias caused by the distribution of the genomes represented, the GIs in one genome from each species and the GIs of the most frequent species are also analyzed. Overall, the results showed that there are preferred sites for the GIs in the genome. In the linear genomes, these sites are usually located in the oriC region and terminus region, while in the circular genomes, they are located solely in the terminus region. These results also showed that the distance distribution between the GIs is almost exponential, which proves that GIs have preferred sites within genomes. The oriC and termniuns are preferred sites for the GIs and a possible natural explanation for this could be connected to the content of the oriC region. Moreover, the content of the GIs in terms of its protein families was studied and the results demonstrated that the majority of frequent protein families are close to identical in each section.

## Introduction

Horizontal gene transfer (HGT) is a process that is common among microbes for obtaining novel genes that are exchanged between species that may not be related taxonomically [1–3]. This process is viewed as a means to quickly adapt to changes in environmental conditions, where the obtained DNA fragments are often of a sizable length [4]. These DNA fragments are generally known as genomic islands (GI) [5], or, more specifically, symbiotic islands [6] or pathogenicity islands [7, 8] based on their content. The GIs can be located in numerous sites within the prokaryotic genome. The location of GIs frequently correlates with noticeable structural features such as mobility genes and tRNA genes, which has led to a definition of the GI structure that incorporates these features [7, 9, 10]. Furthermore, prior studies examining insertion sites for GIs show that tRNA genes are often targeted. In addition, for some instances, the targets are either the intergenic region, or at the 5 or 3 end of protein-coding genes [11–13]. Despite the identification of the most common locations for GIs not being previously investigated, numerous research papers have put forth structural definitions for GIs based on common features [14] which can be used to facilitate the detection of the GIs’ location within the genome. This can be used to facilitate the detection of the GIs’ location within the genome. However, the identification of the most common locations for GIs had not previously been investigated.

Motivated by this lack of information, the main contribution of this paper is to initiate an investigation about GIs from a perspective that has yet to be addressed extensively, which is their most commonly located sites in the genome. This investigation provides a deeper understanding of where the GIs are more frequently located within prokaryotic genomes and was performed using three different analysis techniques. The first technique was to analyize the GIs location in relation to the start of the genome, known as the location of the origin of replication (oriC) [15]. For the second technique, an analysis was performed in order to investigate the distances between the GIs within the genome. Finally, in the third technique, an analysis of the location distribution of GIs in the prokaryotic genomes was carried out. Each of the three techniques was performed using three cases according to the data input utilized: first, all of the GIs in the data set; second, the GIs in one genome from each species; and third, the GIs of the most frequent species in the data set. Furthermore, in each case, the GIs have been divided into two groups, the circular group and the linear group, according to the genome structure where the GI is located. The genome structure is considered here, since we assumed that each genome structure could lead to a different localization strategy of the GIs, and this is in fact what the analysis showed. All of this categoriziation in the analysis was performed to develop the results from different perspectives and to avoid biases. The database resource for all of the genomes and their GIs that has been used in this research is from the IslandViewer database [16]. IslandViewer is a computational tool that integrates four different genomic island prediction methods: IslandPick, IslandPath-DIMOB, SIGI-HMM, and Islander. Using this tool, we retrieved the GIs that were predicted from different methods and each method used a different technique. For example, Islander and IslandPath both detected GIs using the tRNA genes in the genome, meaning that part of our dataset contained GIs that had been detected using the tRNA genes. The investigation was performed on this dataset in order to gain knowledge of the preferred sites of prokaryotic GIs, and this likely has advantages in facilitating the implementation of GI detecting tools as well as improving their accuracy.

## Materials and methods

### Data set

The prokaryotic data set used in this research is the same as the data set used in our prokaryotic patterns detection paper [17], which is from the IslandViewer4 website and is composed of genomes along with the GIs and their proteins. See that paper for details of obtaining the data. During the study, thousands of bacterial genomes were retrieved from IslandViewer. However, we did not differentiate in any way between genomes in terms of content, such as between AT-rich and GC-rich genomes. Furthermore, the database we used for our data source only included the primary chromosomes, and the secondary genomic elements were outside the scope of our study. However, we did differentiate between circular and linear chromosomes to investigate more about the relationship between genome structures and the locations of GIs. In addition to this, we would like to mention that most bacteria appear to have a single large circular chromosome, but this is not universal.

### Problem statement

Our research aims to study the structures of GIs within prokaryotic genomes. This is achieved by analyzing the GIs to understand their location features from different perspectives. In the data set, there are a number, *N*, of genomes where each genome *G*_*i*_ contains a number of GIs *n*_*i*_. One convenient representation of these GIs in *G*_*i*_ is via an ordered sequence, as follows,

$$\begin{array}{c} G_{i}=I_{i,1},I_{i,2},\ldots ,I_{i,n_{i}}, \end{array}$$ (1)

where *I*_*i*,*j*_ is the *j*th island within genome *G*_*i*_. While two islands from the IslandViewer4 data set might overlap, we enforced no overlap by merging any islands that originally overlapped; see below.

### Methods

This section presents the analysis that has been performed on the GIs in order to discover the nature of the locations of GIs within a genome. At the beginning, the GIs reformulated to overcome the overlap issue, then the analysis, as mentioned in the problem statement, was performed. The input of the analysis is the GIs and these GIs were divided into circular group and linear group according to the genome structure that they belong to. Therefore, the three step analysis was performed on each group of GIs separately. Furthermore, each of the three analyses was performed using three cases according to the data input utilized: first, all of the GIs in the data set; second, the GIs in one genome from each species; and third, the GIs of the most frequent species in the data set. The latter two cases were performed to avoid bias, since, within the data set, there are more GIs for a few specific species than for others. To understand the broad view of the GIs, a brief analysis of the GIs in the data set is provided in the SI.

#### The forming of genomic islands

**Fig 1:**
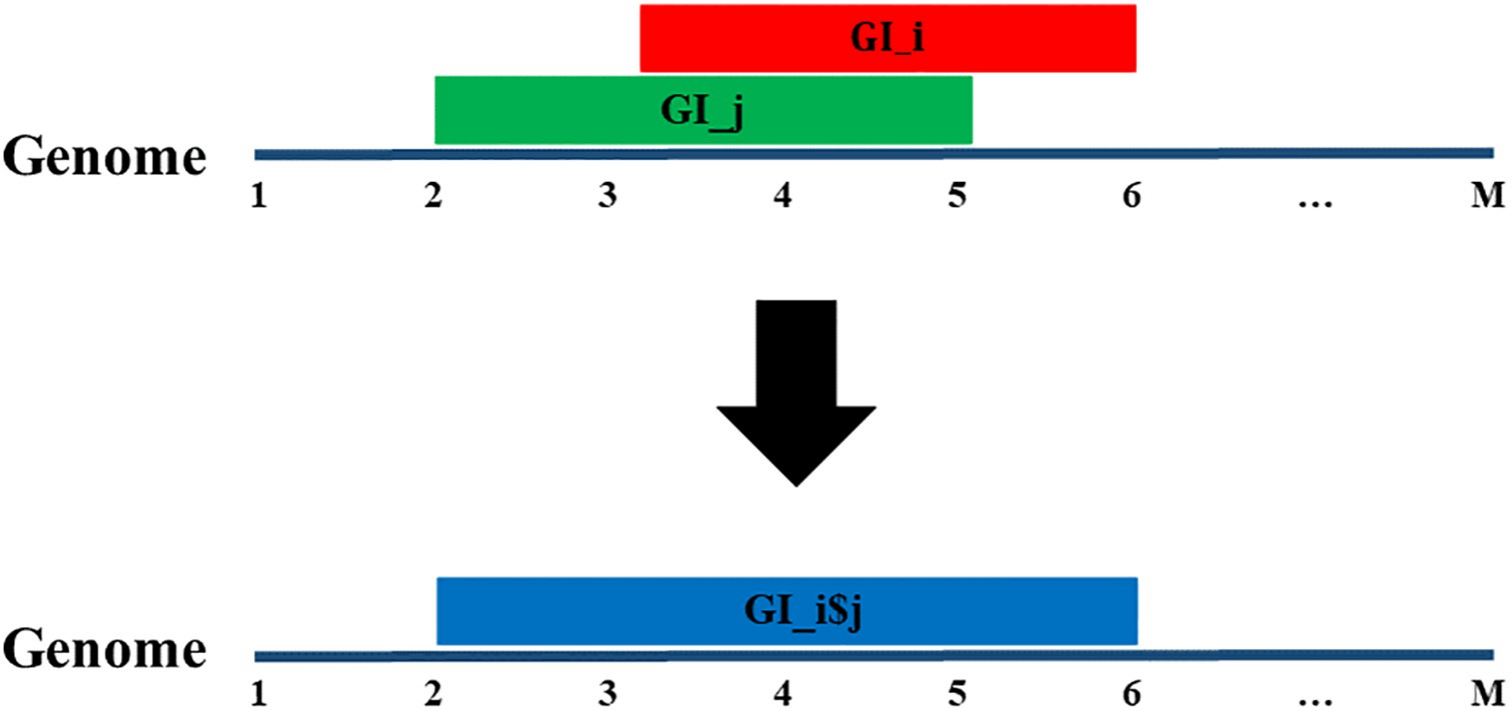
Genomic island overlap.

The data set of GIs used in this research from the IslandViewer4 database is predicted by different tools. Therefore, some of the GIs overlap, whereas others do not. The overlapped GIs are GIs predicted by different tools and share roughly the same location within the genome. However, the start and the end coordinates from the overlapped GIs can be different. In this section, the overlapped GIs have been merged to form a new GI that covers all of the area where the overlapping occurs. The merge was performed to avoid the redundant GIs that are predicted from different GI prediction tools. Moreover, since this part of the genome was determined as a GI by several GI prediction tools, it is more practical to deal with one GI, as all of these GIs share roughly the same area. However, the start and the end can be different for these overlapped GIs since different ones are predicted by different GI prediction tools. Therefore, the overlapped and non-overlapped areas of these GIs form a big GI, starting with the smallest coordinate out of all of the starting points, from the GIs in question and ending with the largest coordinate out of all of the ending points from the GIs as shown in Fig 1.

Moreover, in the S1 File, *The Forming of Genomic Islands* algorithm shows the strategy followed to form these GIs. The total number of the GIs before the overlap equates to 368,339, whereas after the overlap it equates to 257,050. It is worth mentioning that the merge was used instead of the intersection for two reasons. First, the GIs were detected by tools from published journals and, therefore, each GI is worth studying. Second, the intersection could have resulted in very small GIs that may not have produced valuable results. Therefore, the decision that was made to merge could have led to large GIs but it was better than losing valuable areas of GIs.

#### Genomic islands location in relation to the origin of replication

The prokaryotic GIs are in numerous locations within the genome and their preferred location is still unknown. In this research we discovered a feature of these GIs locations by knowing their location in relation to the the location of the origin of replication. This was performed by using the starting point of the GIs to refer to the location of the GI in relation to the location of the oriC. The location of GIs were computed using *The Location of the GIs in Relation to the oriC* algorithm that is provided in the S1 File.

#### The nature of the distances between the genomic islands

**Fig 2:**
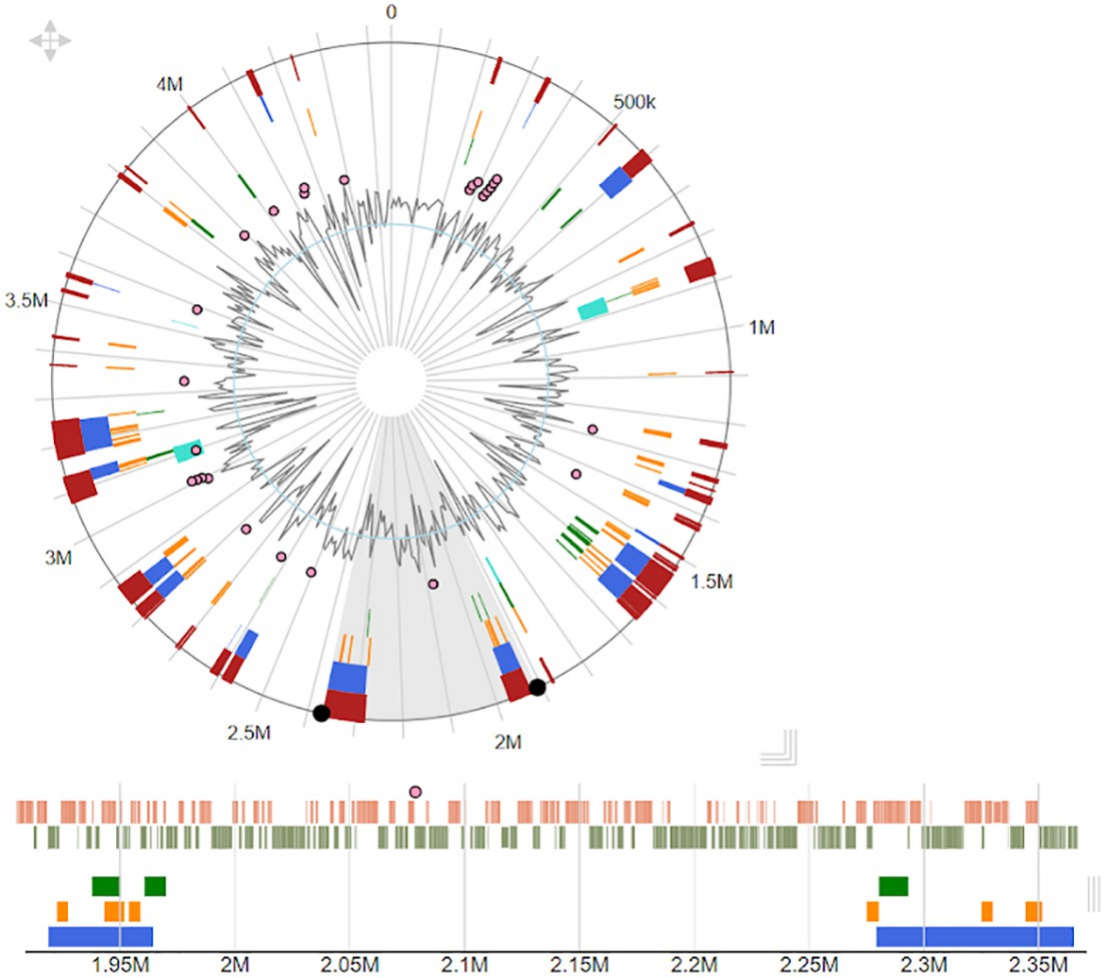
Complete genome of the Acidovorax. The circle shows the complete genome of the Acidovorax sp. (strain JS42) and the resulting GIs that come from IslandPick (Green), SIGI-HMM (Orange), and IslandPath-DIMOB (blue) [16]. The horizontal plot shows the part of the genome shaded in gray between the two black dots in the lower part of the circle. The blue triangle represents the GIs predicted by the IslandPath-DIMOB method, and the rest of the GIs are represented by the color of the predicted method. The distance between GIs is the length of space between every two GIs.

In this section, one of the objectives of the research is to understand the nature of the distance between the GIs. This is performed by normalizing the coordinates of GIs then computing the distance between the GIs. The aim here is to plot the distances to discover if the GIs are located systematically or randomly. In the S1 File, the *Compute the Distance between the GIs* algorithm was used to compute the distances between the consecutive GIs in each genome. The distance between GIs is the length of space between every two GIs, as shown in Fig 2. For example, if we have GI_X and GI_Y, the distance between them starts at the ending point of GI_X and ends at the starting point of GI_Y. This strategy in computing the distances was applied to the GIs that belong to circular and linear genomes. In circular genomes, the distance between the last GI in the genome and the first GI in the genome was also computed.

#### Location distribution of genomic islands in the genome

The prokaryotic GIs are distributed in the genome over different locations. In this section, the locations of the GIs is studied from a different perspective by dividing the genome into n sections or slices and studying the GIs density in each section. The *Distribution of the GIs* algorithm has been used in this step of the research to perform the analysis, which is provided in the S1 File. This algorithm divides the genome into n sections then computes the number of the GIs in each section. A GI is considered located in a section if part of this GI is exists/located in this section.

## Results

This section presents the investigation results of the locations of GIs using the three different analysis techniques.

### Genomic islands location in relation to the origin of replication

**Fig 3:**
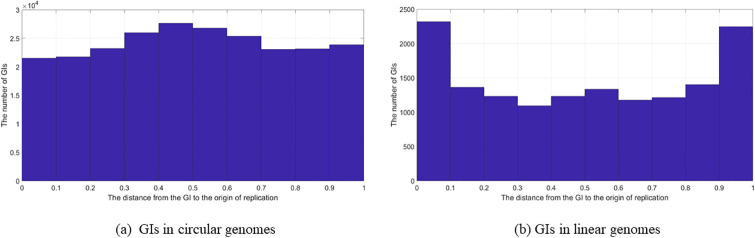
GIs location in relation to oriC (All GIs). The location of the GIs in relation to the oriC using all of the GIs in the data set; (a) GIs in circular genomes. (b) GIs in linear genomes.

The location of the GI in relation to the location of the oriC was plotted as a histogram using Matlab, as shown in Fig 3, where Part (a) of the figure represents all of the GIs in the data set that were located in circular genomes, and Part (b) represents all of the GIs in the data set that were located in linear genomes.

**Fig 4:**
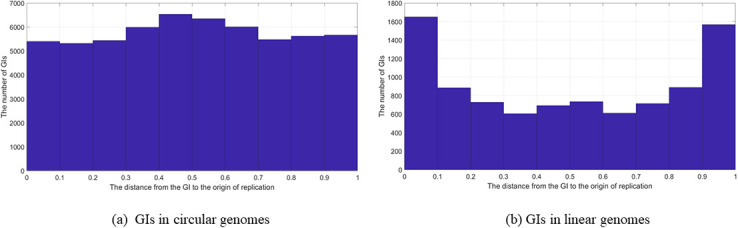
GIs location in relation to oriC (Species). The location of the GIs in relation to the oriC using the GIs in one genome from each species in the data set; (a) GIs in circular genomes. (b) GIs in linear genomes.

Fig 3(a) shows that there is a slight elevation in the terminus. In Part (b), GIs in liner genomes, there is more presence of the GIs at start and the end points (i.e, in the origin and terminus). Fig 4 represents the analysis of the GIs from each species. The result of this analysis showed the same observations as in the “all the GIs” case in Fig 3.

**Fig 5:**
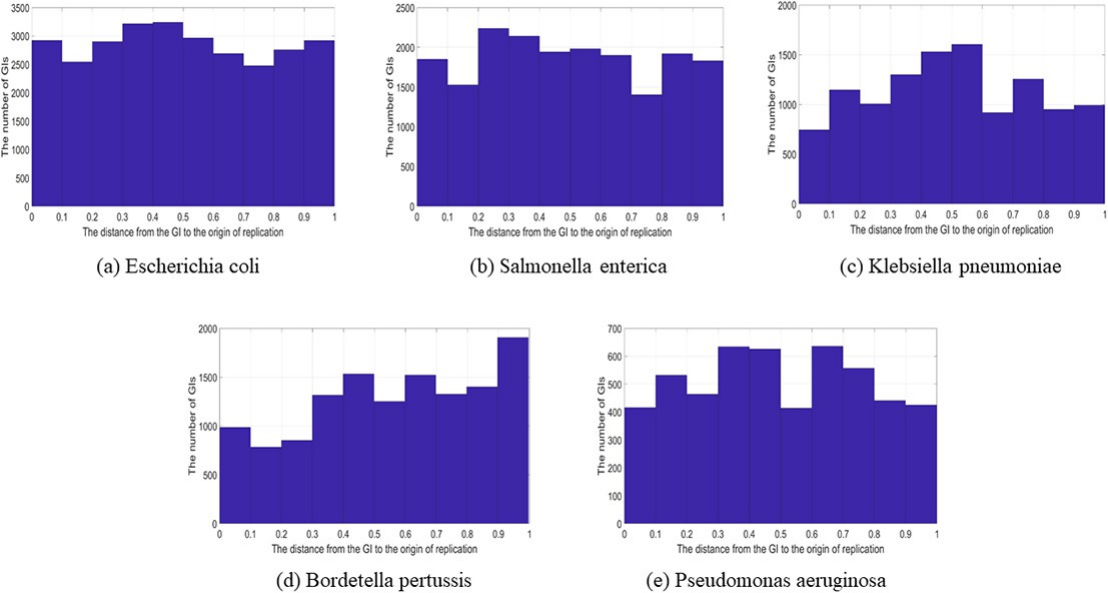
GIs location in relation to oriC (Most frequent species). The location of the GIs of the most frequent species in the data set in relation to the origin of replication in circular genomes; (a) Escherichia coli. (b) Salmonella enterica. (c) Klebsiella pneumoniae. (d) Bordetella pertussis. (e) Pseudomonas aeruginosa.

As mentioned in our previous work [17], the top species in the data set which make up the largest proportion of the data set are *Escherichia coli*, *Salmonella enterica*, *Klebsiella pneumoniae*, *Bordetella pertussis*, and *Pseudomonas aeruginosa*. A histogram was built for each of the top species in the data set that reflect the true location of the GIs in relation to the oriC. This was carried out with no bias because the histogram represents only the GIs in a specific species. Fig 5 shows the histograms of the top five species in the data set.

This analysis was performed for the GIs in circular genomes since, in the data set, very few GIs are located in linear genomes that belong to the most frequent species. However, the analysis for the GIs in linear genomes is provided in the S1 File. Starting with the *Escherichia coli*, there is more presence of the GIs in the terminus and at the end points (i.e., oriC). In *Salmonella enterica* there is a high presence of GIs in the terminus and oriC. In both species, there are few GIs in the genome between 0.1 and 0.2 and between 0.7 and 0.8. The *Klebsiella pneumoniae* histogram shows a high presence of the GIs in the terminus, unlike the other top species that show that their difference in location is not big. In the *Bordetella pertussis* histogram, the presence of GIs generally increase at the end of the chart. There is less of a presence of GIs between 0.1 and 0.2. Finally, *Pseudomonas aeruginosa* shows a high presence in the terminus, where there is a slight drop that is located in the genome between 0.5 and 0.6. The Kolmogorov-Smirnov uniformity test was used to investigate the distribution of the GIs in relation to the oriC in all of the figures mentioned above, whether it is a uniform distribution or not. In statistics, a uniform distribution means every possible result (i.e. distance) has an equal chance of occurring. Table 1, shows that all the figures do not follow the uniform distribution since all the p-values are less than 0.05; therefore, this leads to rejecting the null hypothesis. The null hypothesis is rejected if the p-value is at the 5% significance level or less.

**Table 1:** Uniformity test for the GIs location in relation to the oriC.

GI Category	P-value
All the GIs	1.405e-145
Genome from each species	1.021e-24
Escherichia coli	1.993e-15
Salmonella enterica	1.939e-20
Klebsiella pneumoniae	3.002e-26
Bordetella pertussis	1.749e-137
Pseudomonas aeruginosa	1.406e-09

**Fig 6:**
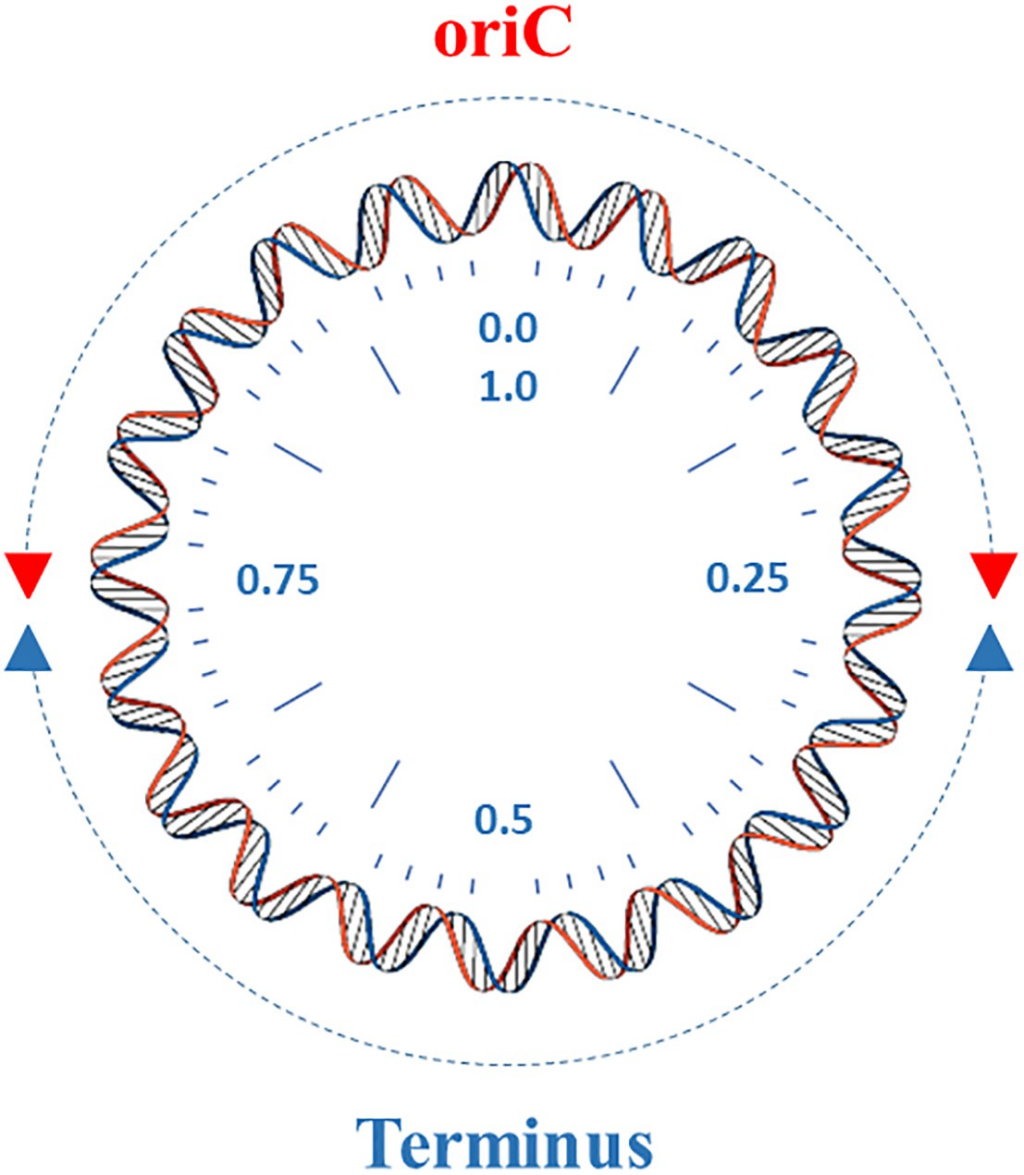
Circular genome. A circular genome divided into two equal size arcs; oriC arc (from 0.0 to 0.25 and from 0.75 to 1.0) and terminus arc (from 0.25 to 0.75).

Overall, there are different places, as shown in the diagrams mentioned above, containing GIs, but generally they are concentrated in the terminus or the oriC region. Regarding the terminus, the density of GIs in circular genomes is more in the terminus than in other areas. Consequently, an investigation was performed in order to investigate further about the density of the GIs in the terminus and the oriC region. In this analysis step, the circular genomes were divided into two equal sections (i.e., arcs), as shown in Fig 6. GIs in the area from 0.0 to 0.25 or 0.75 to 1.0 are considered located in the oriC region. Furthermore, GIs located between 0.25 and 0.75 are considered to belong to the terminus region.

The number of the GIs were counted in both areas, as shown in Table 2. As shown in the table, in all the GI types, the number of GIs in the terminus is more significant than in the oriC region.

**Table 2:** The number of GIs in circular genomes, in the oriC region and the terminus, for each data input utilized.

GI Category	oriC	terminus
All the GIs	114,025	128,408
Genome from each species	27,482	30,335
Escherichia_coli	14108	14516
Salmonella_enterica	9330	9400
Klebsiella_pneumoniae	4968	6476
Bordetella_pertussis	6142	6734
Pseudomonas_aeruginosa	2239	2906

**Fig 7:**
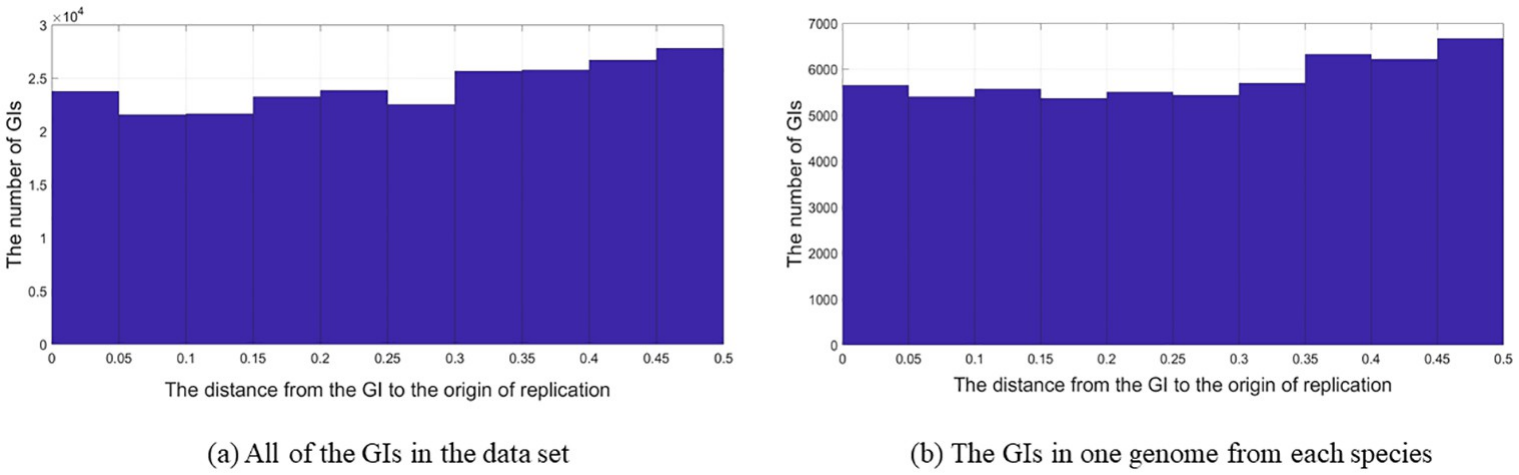
Circular genomes with a range of 0 to 0.5. The location of the GIs in relation to the origin of replication in circular genomes with a range of 0 to 0.5; (a) All of the GIs in the data set, (b) The GIs in one genome from each species.

**Fig 8:**
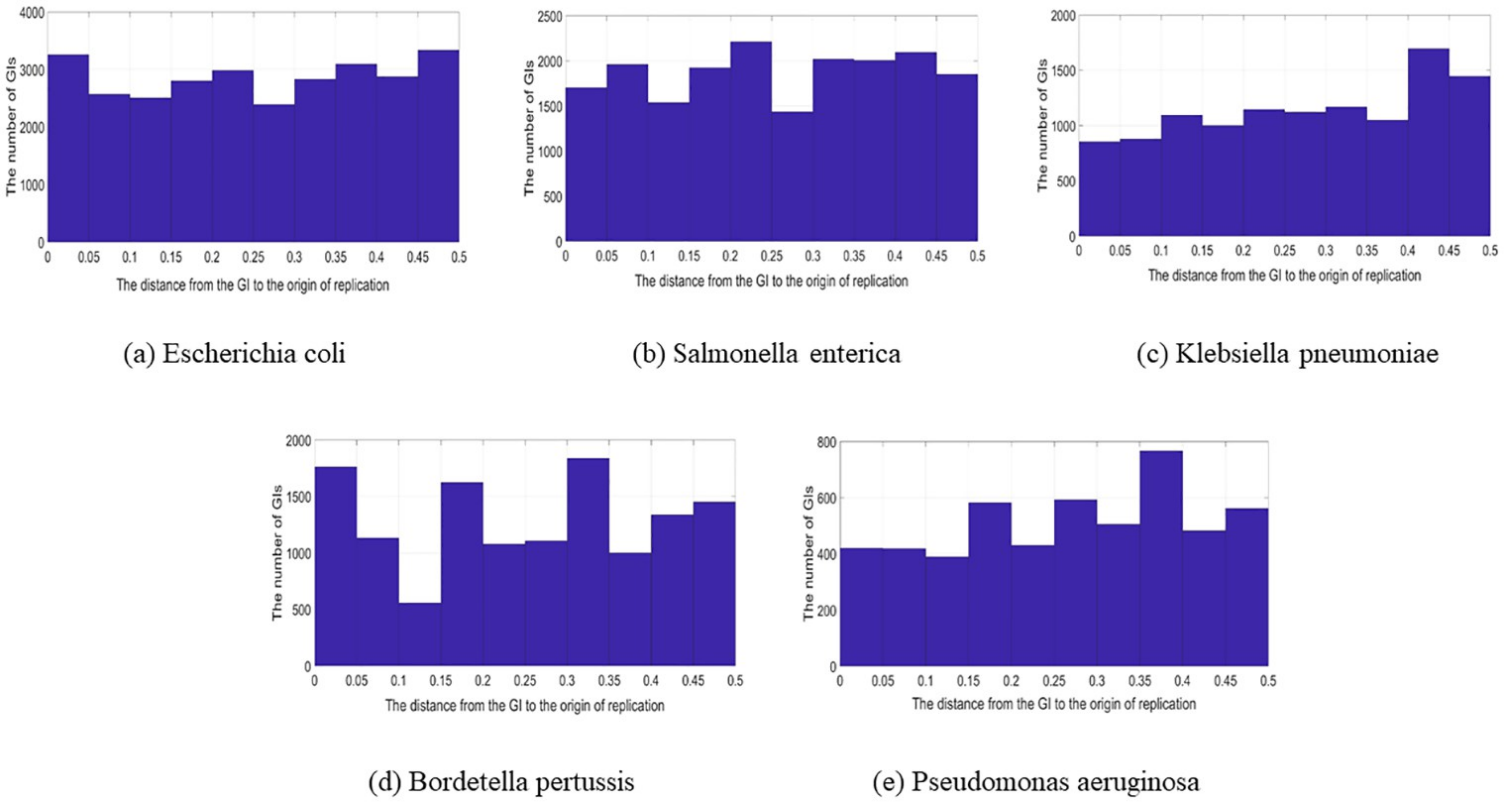
Circular genomes with a range of 0 to 0.5 (Most frequent species). The location of the GIs in relation to the origin of replication in circular genomes ranges from 0 to 0.5; (a) Escherichia coli. (b) Salmonella enterica. (c) Klebsiella pneumoniae. (d) Bordetella pertussis. (e) Pseudomonas aeruginosa.

This also can be seen in Figs 7 and 8. More precisely, in circular genomes, a GI that is 0.1 away from the oriC (oriC locus = 0.0) is the same as a GI 0.9 from the oriC. Therefore, at this step, the x-axis scale in Figs 3–5 was changed from (0 to 1) to (0 to 0.5). This was performed by subtracting any GI located in a locus greater than 0.5 by 1, and keeping the other GIs the same if they are located between 0 and 0.5. For this reason a GI located between 0 and 0.25 are in the oriC region, and GIs between 0.25 and 0.5 are in the terminus region.

### The nature of the distances between the genomic islands

**Fig 9:**
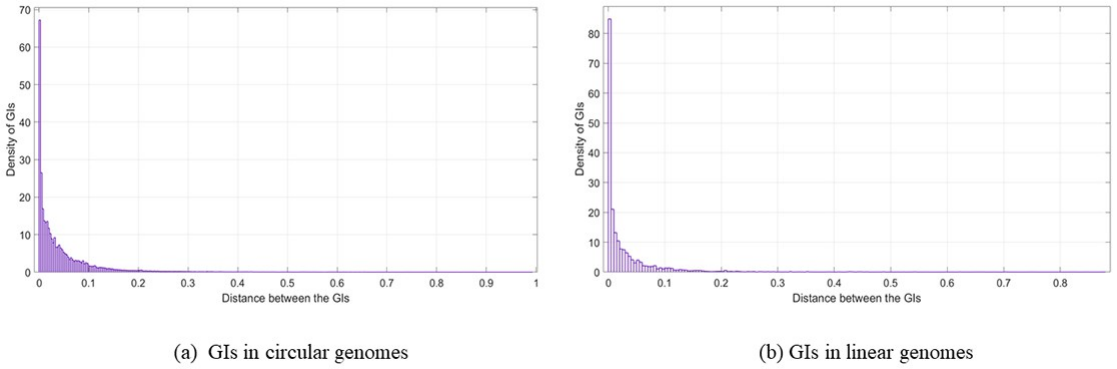
The distance between the GIs using all of the GIs in the data set. (a) GIs in circular genomes. (b) GIs in linear genomes.

**Fig 10:**
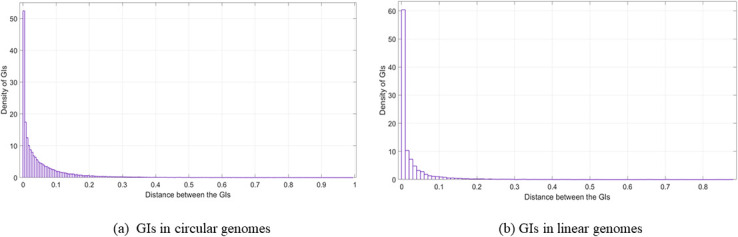
Distance between the GIs (Species). The distance between the GIs using the GIs in one genome from each species in the data set; (a) GIs in circular genomes. (b) GIs in linear genomes.

**Fig 11:**
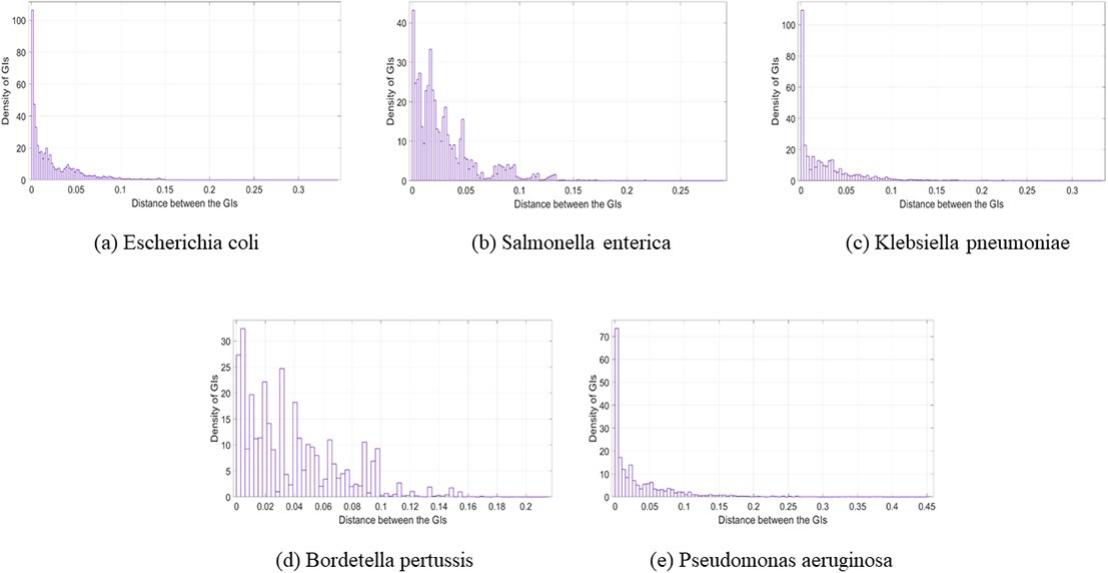
Distance between the GIs (Most frequent species). The distance between the GIs in the most frequent species in the data set (Circular genomes); (a) Escherichia coli. (b) Salmonella enterica. (c) Klebsiella pneumoniae. (d) Bordetella pertussis. (e) Pseudomonas aeruginosa.

In this section, the resulting distances was plotted using Matlab. The distribution fitter application from the statistical and machine learning toolbox in Matlab was used to depict the distances. Moreover, the parameter Log likelihood was used as a measure of selecting the distribution, where the higher the value was, the better. Overall, the resulting distance charts look almost like an exponential probability distribution for most of the charts, as shown in Figs 9–11. From these charts, it is obvious that most of the GIs are usually close to each other and this supports the previous results in the GIs location in relation to the origin of replication section where there is high presence of the GIs in relation to the oriC in specific locations in the genome. In this part of the analysis, the idea of using a histogram was to condense the data series into an easily interpreted visual by taking many data points and grouping them into logical ranges. In our data set, as an example, the number of GIs belonging to *Escherichia coli* is very large compared to the number of GIs in *Salmonella enterica* and *Bordetella pertussis*. For this reason, small values in the histogram will not be seen in Figs 9 or 10. The analysis for the GIs in linear genomes is provided in the S1 File.

### Location distribution of genomic islands in the genome

**Fig 12:**
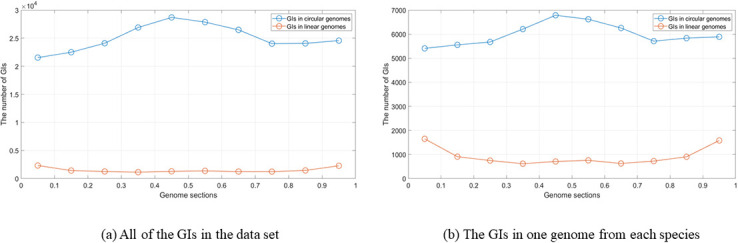
The distribution of GIs. Using (a) All of the GIs in the data set. (b) The GIs in one genome from each species.

**Fig 13:**
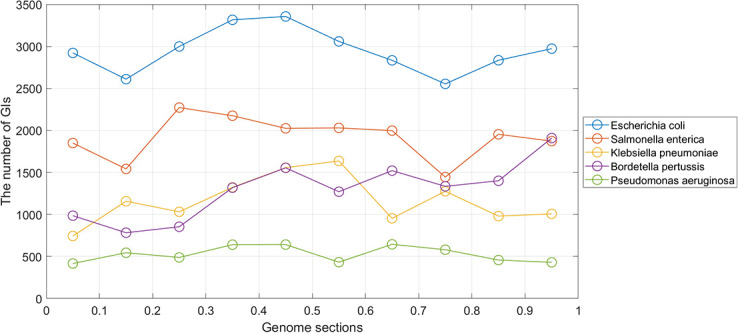
The distribution of GIs using the most frequent species in the data set.

The results of the previous two steps analysis proved that there were specific locations in which GIs are abundant. Therefore, in this section, we investigate more about these findings by studying the location distribution of GIs in the genome. In the location distribution of GIs in the genome analysis, the analysis was performed on the GIs for n = 10. This means each genome was divided into ten equal parts then the number of the GIs in each section was computed. This analysis aimed to know the location of the GIs more precisely in the terminus and oriC region. Fig 12(a) shows the distribution using all of the GIs in the data set. In the figure, there are two lines; the blue line represents the GIs that are located in the circular genomes, whereas the orange line represents the GIs that are located in the linear genomes. Regarding the GIs in the circular genomes, it is clear that there is a notable elevation in the terminus. In regards to the GIs in linear genomes, overall, there is no significant difference but there is a slight elevation in the oriC and terminus. In Fig 12(b), the analysis of the GIs from each species shows the same results as in the “all the GIs” case. The only difference is that in the GIs in the linear genomes, the elevation in the oriC and terminus is more evident than in the “all the GIs” case. The analysis was also performed on the most frequent species in the data set; *Escherichia coli*, *Salmonella enterica*, *Klebsiella pneumoniae*, *Bordetella pertussis*, and *Pseudomonas aeruginosa*. Fig 13 shows the most frequent species GIs distribution for circular genomes. The analysis for the GIs in linear genomes is provided in the S1 File.

In *Escherichia coli*, the elevation of the GIs is in the terminus and the oriC region. For the *Salmonella enterica*, the density of the GIs in the genome is in locus between 0.2 and 0.3. Regarding the *Klebsiella pneumoniae*, the GIs are more located in the terminus in the locus between 0.5 and 0.6. The *Bordetella pertussis* GIs are more located at the end between 0.9 and 1. Finally, the *Pseudomonas aeruginosa*, the distribution of the GIs is almost uniform. It’s worth mentioning that there is less occurrence of the GIs in two locations; between 0.1 and 0.2, and between 0.7 and 0.8, and this can be seen in the GIs of *Escherichia coli* and *Salmonella enterica* species. The content of the GIs in terms of protein families was studied and the results showed that the most frequent protein families are almost identical in each section. The chart is provided in the .

## Discussion

Horizontal gene transfer (HGT) is a strong source of genetic and physiological alterations that take place within bacteria. This is the main motivation that prompted us to search to discover where the significant elements (i.e. GIs) that facilitate the HGT are usually located within genomes. In our research, the locations of GIs in prokaryotic genomes were investigated from different perspectives to locate GIs preferred sites within the genome, focusing on the density of the GIs. The GIs data set was detected from various tools, which could have led to duplicate findings. To overcome this, GIs were preprocessed to eliminate redundancy or overlaps. In the analysis, we conducted separate analyses for circular genomes and for linear genomes in order to avoid bias. For each group, the analyses were initially performed on all of the GIs in the dataset. Then, in order to see the results from different perspectives, we refined the analysis by studying one GI from each species genome. After that, we prioritized studying the most prevalent bacterial species in the data set. Furthermore, the data set was analyzed using three techniques: the first was based on oriC; the second on the distances between GIs; the third on the distribution of GIs. As mentioned previously, the concept of GI location in relation to the oriC section is different from that of the distribution of GIs section. Consequently, varying results between the two analyses could arise, or the two analyses can support each other. The length of the GIs is one of the factors that may lead to concordance or disparity in the analysis outcomes. In our case, the results of the two analyses supported each other. Overall, all analyses indicated that the presence of GIs may have been at any location in the genome, however, there was a higher concentration of GIs in the terminus region and oriC region. As it is well-known, HGT can occur widely in the terminus region and oriC, where replication and recombination processes converge and, generally speaking, may potentially influence or regulate HGT events in bacteria [18, 19]. In our analysis, the focus was specifically on the GIs presence within the genome. More specifically, we wanted to distinguish between linear and circular genomes and where the highest density of GIs was located. As a result, we discovered that the terminus is a preferred site for the GIs to reside within the circular genomes, while the oriC and terminus are both preferable locations for the GIs in linear genomes. The frequent presence of GIs in terminus and oriC has not yet been investigated extensively in the literature. The high presence of GIs in the oriC region or terminus region could be due to many factors that require deep biological investigation and can be investigated further in future studies. This paper is more focussed on the occurrence and distribution of GIs without investigating the biological side. James P. J. Hall et al. stated that HGT entails two physical processes in principle. The initial process involves genetic information having to cross biological membranes into the recipient species, while the second involves genes being linked to a functioning origin of replication in a germ line cell, ensuring vertical transmission in the recipient [18]. It is also important to mention that despite GC content being found to vary only a small amount within prokaryotic genomes, some regions can differ more than other regions [20]. For example, a large region flanking the replication origin is more GC rich than the typical genomic GC content [21], while the region surrounding the replication terminus is more AT rich [22]. One of our analysis findings showed that the most frequent protein families are almost identical in each section. For this reason, and because the literature shows an investigation has been performed to understand HGTs contributions to protein families growth in prokaryotic genomes [23], it is suggested, as a future work, to perform the investigation at the protein-family level or, more specifically, at the protein level. In general, genome content is crucial for understanding HGT patterns in bacteria, as both the oriC and the terminus region play significant roles in facilitating HGT. Therefore, investigating why the terminus and oriC regions are preferred locations could lead to discovering the characteristics of these regions of the genome in terms of proteins or protein families that make them preferable sites for GIs.

## Supporting information

S1 File (ZIP)

S1 Fig (TIF)

S2 Fig (TIF)

S3 Fig (TIF)

S4 Fig (TIF)

S5 Fig (TIF)

S6 Fig (TIF)

S7 Fig (TIF)

S8 Fig (TIF)
