# Supplementary material for: Investigating the nature of prokaryotic genomic island locations within a genome
Source: PLoS One. 2024 May 2;19(5):e0301172. doi: 10.1371/journal.pone.0301172 (PMC11065298; doi:10.1371/journal.pone.0301172)
Supplement: S1 File — (ZIP) [file pone.0301172.s001.zip › S1_File.pdf]

Supplemental Document for Investigating the Nature of Prokaryotic Genomic Island Locations within a Genome

Reem Aldaihani
Department of Computer Science
Kuwait University, State of Kuwait
Virginia Tech, Blacksburg, VA

Lenwood S. Heath
Department of Computer Science
Virginia Tech, Blacksburg, VA

April 9, 2024

Contents

A Genomic Islands Analysis **2**

B Algorithms **4**

C Location Distribution of Genomic Islands in the Genome (Protein families) **8**

D Linear genomes **9**

A Genomic Islands Analysis

In this section, the GIs are briefly analysed in order to understand the broad view of the GIs in the data set. In the first step of the analysis, the aim is to discover the number of the GIs in the genomes by understanding what the maximum number of GIs in a genome is as well as the minimum number of GIs within a genome. Furthermore, the aim is to discover the length of the genomes and GIs in the data set.

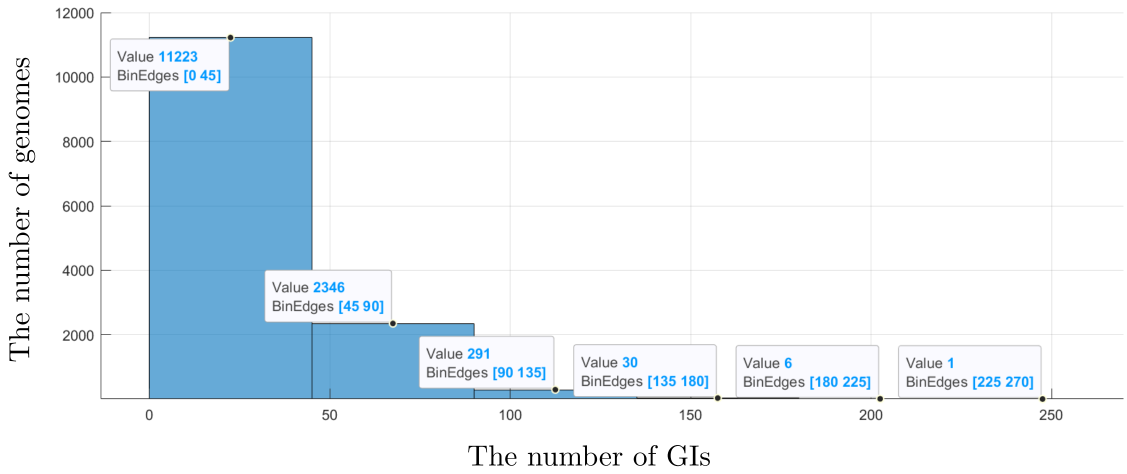

Figure S(1) –The number of the GIs in the genomes

The histogram in Figure 1 answers the first aforementioned question. As shown in Figure 1, the x-axis demonstrates that the range of the GIs is from 1 to 270 in the genomes, whereas the y-axis demonstrates the number of genomes. This means the minimum number of GIs in the genome is one, while the maximum number of GIs in a genome is 270. Overall, it is apparent from the figure that there are many genomes with few GIs, while there are also many GIs in few genomes. For example, the fist bar shows that there are 11,223 genomes that have GIs in the range of 1 and 45. Furthermore, there is only one genome that has a number of GIs in the range of 225 and 270, which is actually the genome NC_011894.1 containing 270 GIs in its structure.

In the data set, the length of the genomes and GIs vary. Therefore, the other step of the GI analysis is to discover the lengths of the genomes in the data set as well as the lengths of the GIs and see how they are related. As shown in Figure 2, most of the genomes in the data set have a length range from $1,510,000$ to $5,940,000$, as shown in the tallest three bars in the middle of the histogram chart. On the other hand, it has been observed that the length of most GIs after normalisation is less than 0.1 as shown in figure 3. This means that most GIs make up approximately 10% or less of the length of the genome.

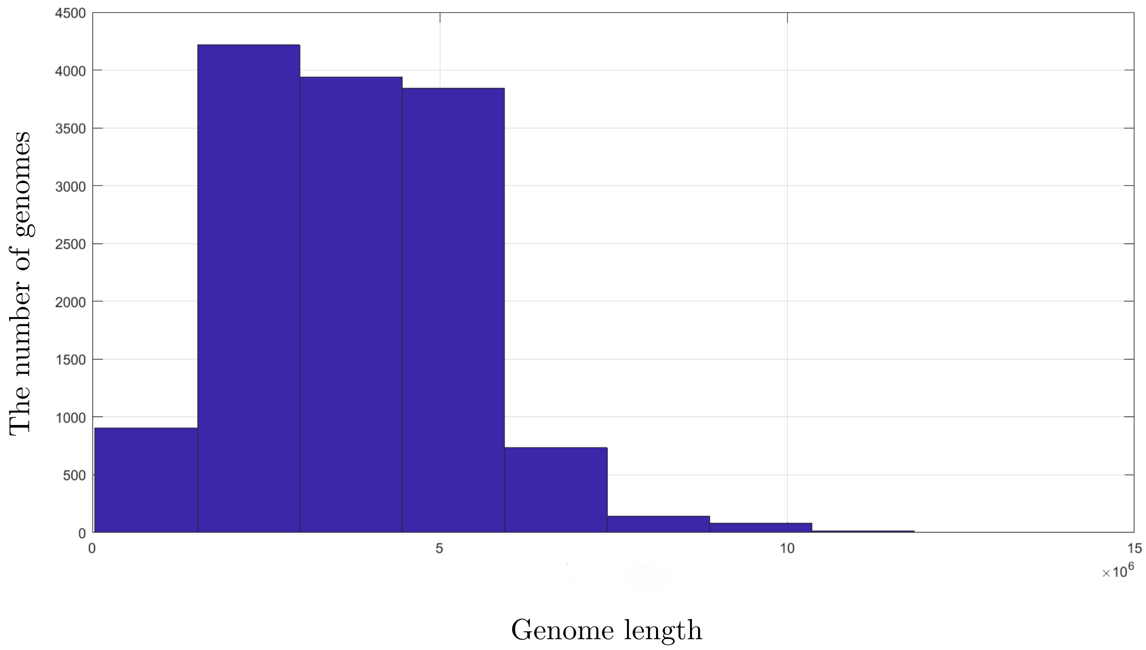

Figure S(2) –The length of the genomes in the data set

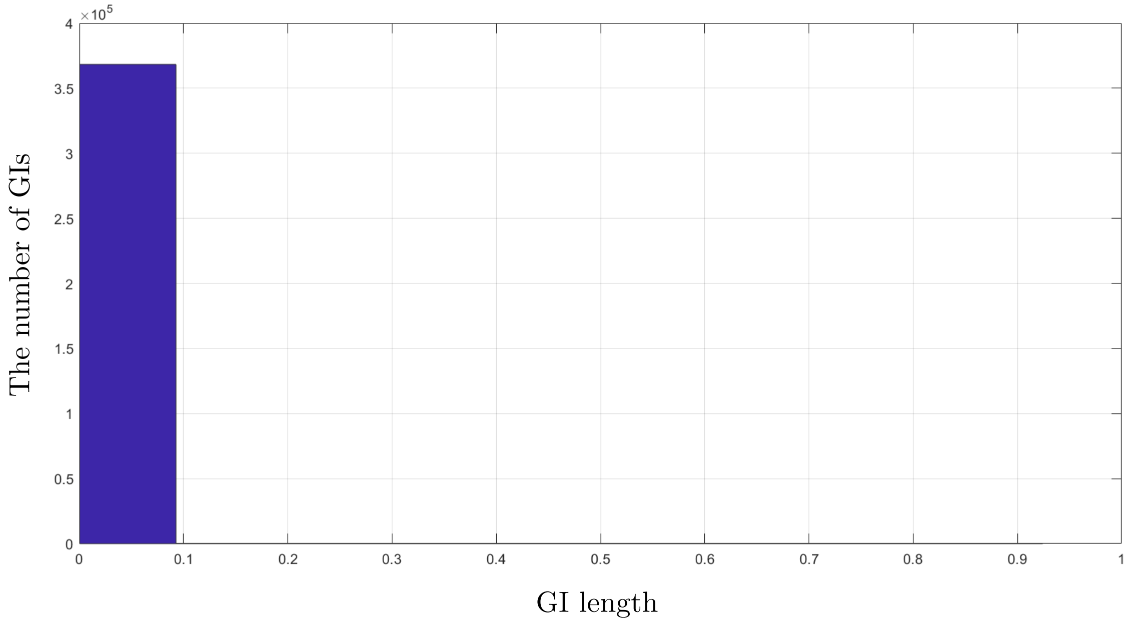

Figure S(3) –The length of the GIs in the data set

B Algorithms

Algorithm 1: The Forming of Genomic Islands

Input: Genomic Islands

Output: The updated version of the genomic islands

```
For each Genome G:
  Sort_GIs_Coordinates() // Sort the GIs in Genome G according to the start point
  GI_Overlap = 0
  For each GI:
    GI = Get_GI_Name
    S = Get_GI_Start_Coordinate
    E = Get_GI_End_Coordinate
    IF GI_Overlap == 0 :
      GI_Overlap = 1,   S_Overlap = S,   E_Overlap = E
    ELSE:
      IF S >= S_Overlap and S <= E_Overlap:
        IF S < S_Overlap:
          S_Overlap = S
        IF E > E_Overlap:
          E_Overlap = E
      ELSE:
        Overlap_GIs.write(GI+ ' ' +S_Overlap+ ' ' +E_Overlap)
        GI_Overlap = 1,   S_Overlap = S,   E_Overlap = E
  IF GI_Overlap == 1:
    Overlap_GIs.write(GI+ ' ' +S_Overlap+ ' ' +E_Overlap)
```

Algorithm 2: The Location of the GIs in Relation to the oriC

Input: Genomic_Islands

Output: The distance from the GIs to the oriC

```
oriC=open('oriC.txt','w')
Species=open('Species.txt','w')
MostFreqSpecies=open('MostFeqSpecies.txt','w')
Species_Circular_Seen=[]; Species_Linear_Seen=[]
MostFreq_Species=['Escherichia coli', 'Salmonella enterica', 'Klebsiella pneumoniae', 'Bordetella pertussis',
'Pseudomonas aeruginosa']
Middle=0; Oric=0; Species_Middle=0; Species_Oric=0
MostFreq_Middle=0; MostFreq_Oric=0;
For each Genome:
  For each GI in Genomic_Islands:
    G = Get_G(GI)
    S = Get_Start_GI(G,GI)/ Length (G)
    Structure=Get_Structure(GI)
    Species=Get_Species(G)
    IF Genome_Structure == "circular" :
      oriC.write(str(S)+'circular')
      IF 0.25 < Start <= 0.75: Middle = Middle + 1
      ELSE: oriC = oriC + 1
      IF (Species not in Species_Circular_Seen) or (not read all GIs) :
        Species.write(str(S)+'circular')
        Species_Circular_Seen.append(Species)
        IF 0.25 < Start <= 0.75: Species_Middle = Species_Middle + 1
        ELSE: Species_oriC = Species_oriC + 1
      IF Species in MostFreq_Species :
        MostFreqSpecies.write(str(S)+Species+'circular')
        IF 0.25 < Start <= 0.75: MostFreq_Middle.add(Species, 1)
        ELSE: MostFreq_oriC.add(Species, 1)
    ELIF Genome_Structure == "linear":
      oriC.write(str(S)+'linear')
      IF (Species not in Species_Linear_Seen) or (not read all GIs) :
        Species.write(str(S)+'linear')
        Species_Linear_Seen.append(Species)
      IF Species in MostFreq_Species :
        MostFreqSpecies.write(str(S)+Species+'linear ')
```

Algorithm 3: Compute the Distance between the GIs

Input: Genomic Islands, Genomes

Output: Distance between the GIs

```
Distance_GIs=open('Distance_GIs.txt','w')
Distance_Species=open('Distance_Species.txt','w')
Distance_MostFreqSpecies=open('Distance_MostFreqSpecies.txt','w')
Species_Circular_Seen=[]; Species_Linear_Seen=[]
MostFreq_Species=['Escherichia coli', 'Salmonella enterica', 'Klebsiella pneumoniae', 'Bordetella pertussis',
'Pseudomonas aeruginosa']
For G in each Genomes:
  GIs=[]; S_Cr=[]; E_Cr=[];
  Species=Get_Species(G)
  For each GI in G:
    GI=Get_GI(G)
    S = Get_Start_GI(G,GI)/ Length (G)
    E = Get_End_GI(G,GI)/ Length (G)
    GIs.append(GI),   S_Cr.append(S),   E_Cr.append(E)
  //Compute the distances
  count = length(GIs)
  IF Genome_Structure == "circular":
    For i in range (0,count):
      IF i == (count − 1):
        Distance=round(abs( (S_Cr[0]/Genome_Size) + (1-(E_Cr[i]/Genome_Size)) ),5)
      ELSE:
        Distance=round(abs( (S_Cr[i+1]-E_Cr[i])/Genome_Size ),5)
      Distance_GIs.write(str(Distance),'circular')
      IF (G not in Species_Circular_Seen) or (not read all GIs) :
        Distance_Species.write(str(Distance),' circular')
      IF Species in MostFreq_Species :
        Distance_MostFreqSpecies.write(str(S)+Species+'circular')
  ELIF Genome_Structure == "linear":
    For i in range (0,count-1):
      Distance=round(abs( (S_Cr[i+1]-E_Cr[i])/Genome_Size ),5)
      Distance_GIs.write(str(Distance)+' linear')
      IF (G not in Species_Linear_Seen) or (not read all GIs) :
        Distance_Species.write(str(Distance),'linear')
      IF Species in MostFreq_Species :
        Distance_MostFreqSpecies.write(str(S)+Species+'linear')
  GIs.clear(); S_Cr.clear(); E_Cr.clear()
```

Algorithm 4: Distribution of the GIs

Input: Genomic Islands

Output: Distribution of GIs

```
GIs_Distribution=open('GIs_Distribution.txt', 'w')
n=10
    P=[]; a=1/n; sum=0
For i in range (0,n):
    sum+=a
    P.append(round(sum,1))
index=-1; Parts_list_Circular=[]; Parts_list_Linear=[];
For PL in range(0,n):
    Parts_list_Circular.append([]); Parts_list_Linear.append([]);
For i in GIs:
    index+=1
    Genome_IDX=Genome.index(i.split('_B_')[0])
    Genome_Size=int(size[Genome_IDX])
    Genome_Structure=Structure[Genome_IDX]
    Start=S[index]/Genome_Size; End=E[index]/Genome_Size
    Length=abs(End-Start); Prev_j= 0; P_index=-1
    For j in P:
        P_index+=1;
        IF Prev_j < Start <= j:
            IF Prev_j < End <= j:
                IF Genome_Structure == "circular": Parts_list_Circular[P_index].append(i)
                ELIF Genome_Structure == "linear": Parts_list_Linear[P_index].append(i)
            ELSE:
                IF j − Start > 0:
                    IF Genome_Structure == "circular" : Parts_list_Circular[P_index].append(i)
                    ELIF Genome_Structure == "linear" : Parts_list_Linear[P_index].append(i)
                For k in range (P_index,len(P)-1):
                    IF P[k] < End <= P[k + 1]:
                        IF Genome_Structure == "circular": Parts_list_Circular[k+1].append(i)
                        ELIF Genome_Structure == "linear": Parts_list_Linear[k+1].append(i)
                        break
                    ELSE:
                        IF Genome_Structure == "circular": Parts_list_Circular[k+1].append(i)
                        ELIF Genome_Structure == "linear": Parts_list_Linear[k+1].append(i)
        Prev_j=j
For i in Parts_list_Circular:
    GIs_Distribution.write(str(len(i))+'circular')
For i in Parts_list_Linear:
    GIs_Distribution.write(str(len(i))+'linear')
```

C Location Distribution of Genomic Islands in the Genome (Protein families)

In general, most prokaryotic GIs share the same fact that they are abundant in certain areas within a genome. Therefore, it is significant to discover the content of the GIs in these areas that make them preferred sites for GIs. In this section, the content of the GIs (i.e. Protein families) from the most frequent species distribution chart was studied. Figure S(4) shows that the Phage_integrase and rve protein families always exist in each section, and Phage_integrase is the most abundant protein family. After that, in no specific order, come the other protein families; HTH_Tnp_1, HTH_3, ABC_tran, and LysR_substrate. The aforementioned protein families are the most frequent protein families in the GIs in each section in the genome. In general, the results showed that the most frequent protein families are almost identical in each section.

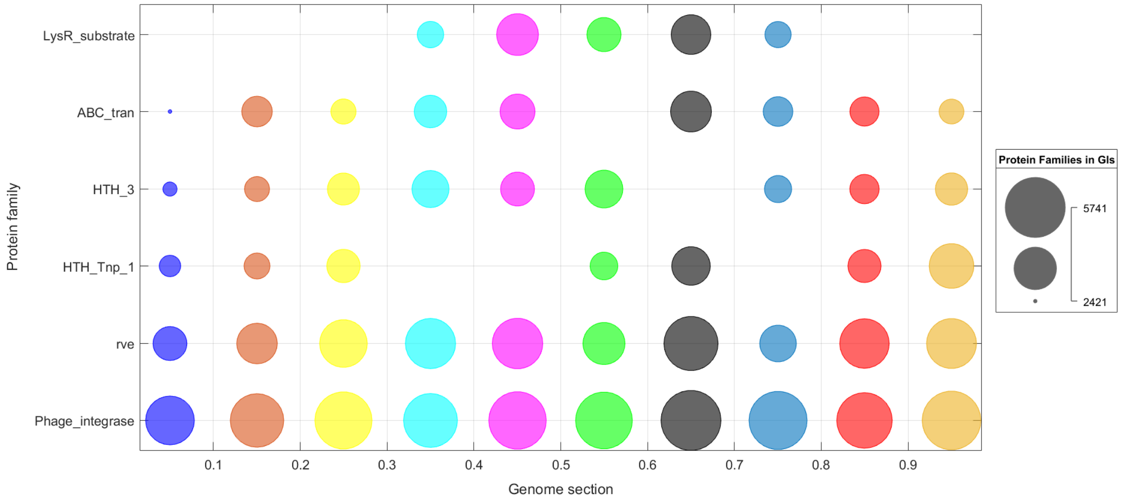

Figure S(4) −The most frequent protein families in each section in the genomes

D Linear genomes

In this section, all of the analyses that were performed for the GIs in circular genomes of the top species were performed for the GIs in linear genomes of the top species. It should be noted that the number of GIs in linear genomes is less than the number of GIs in circular genomes in the data set, and this is due to the small quantity of linear genomes in the data set. Moreover, it must be taken into consideration that the number of GIs here is small, so the results may change and be more accurate when the number of GIs increases. Although it is likely that the results are not completely accurate due to the small number of GIs owing to the small number of linear genomes, we wanted to mention them in order to have an initial vision of the location of GIs in the linear genomes.

Firstly, Figure S(5) represents the GIs' location in relation to the origin of replication (oriC) of the most frequent species in the data set. Starting with the *Escherichia coli*, it's apparent that the number of GIs is almost uniform except in the region between 0.1 and 0.3 and in the range between 0.9 and 1. The difference between these two regions and the other regions is around only five GIs. In *Salmonella enterica*, it can be seen that the number of GIs in oriC is higher than in the terminus, by around 30 GIs. Furthermore, the chart shows that the number of GIs is high in the range between 0.4 and 0.6, and the number of GIs in these regions is higher than the oriC and terminus by around 35 to 40 GIs. In the *Klebsiella pneumoniae* chart, the number of GIs is low where the maximum density of GIs in a region does not exceed 18 GIs. The chart shows a high presence of the GIs in the region between 0.7 and 0.8 (i.e., 18 GIs) and in the region between 0.3 and 0.4 (around 17 GIs). The difference between these regions and most other regions is around 1 to 10 GIs, excluding the regions with very few GIs (i.e., six GIs or less), such as between 0 and 0.1 (approximately) as well as between 0.6 and 0.7. Regarding the *Bordetella pertussis* chart, the general presence of GIs is located in the terminus between 0.9 and 1 with seven GIs. In the other regions, the density of GIs is between three to five GIs, not including the region between 0.8 and 0.9 where there is a drop in the number of GIs.Finally, the *Pseudomonas aeruginosa* chart shows a high presence of the GIs in the region between 0.3 and 0.4 as well as in the region between 0.7 and 0.9.

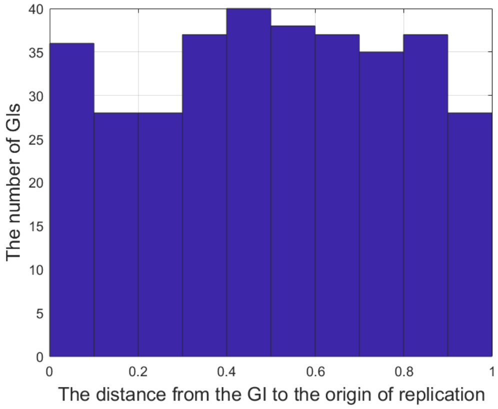

(a) Escherichia coli

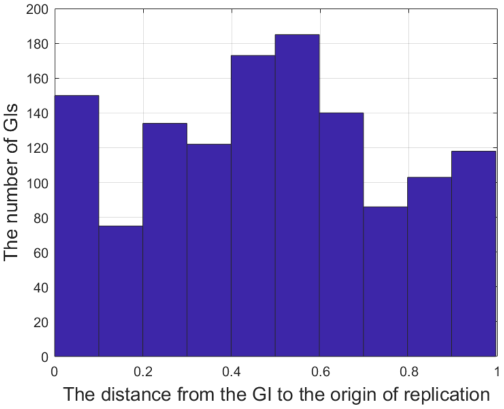

(b) Salmonella enterica

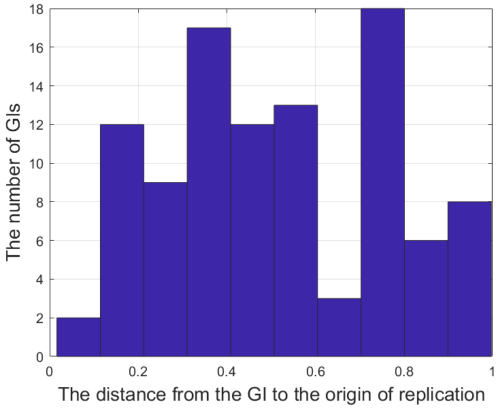

(c) Klebsiella pneumoniae

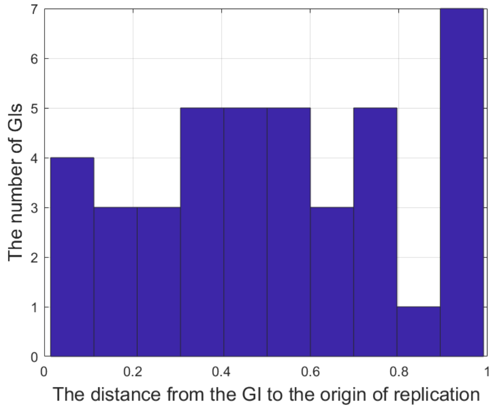

(d) Bordetella pertussis

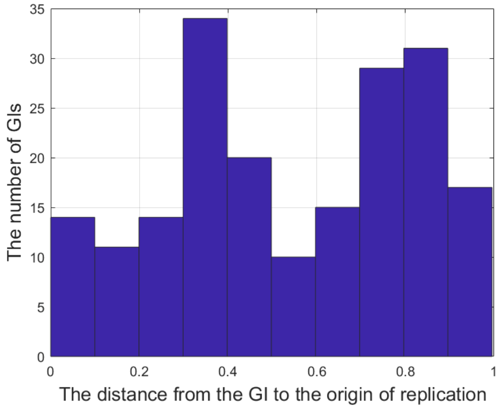

(e) Pseudomonas aeruginosa

Figure S(5) –The location of the GIs of the most frequent species in the data set in relation to the origin of replication in linear genomes

Secondly, Figure S(6) shows the nature of the distances between the GIs in the most frequent species in the data set. Here, we also had the same observation that we had had in the circular genomes, where all charts followed an exponential distribution. This means that GIs are usually close to each other, meaning that there are specific preferred locations of the GIs within the genome.

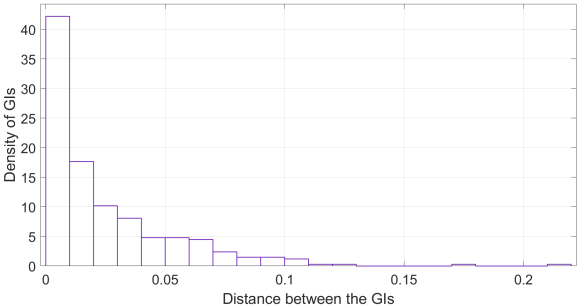

(a) Escherichia coli

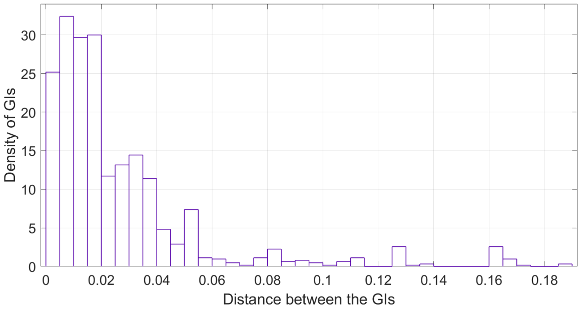

(b) Salmonella enterica

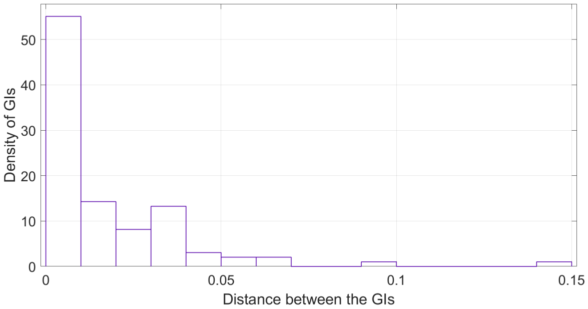

(c) Klebsiella pneumoniae

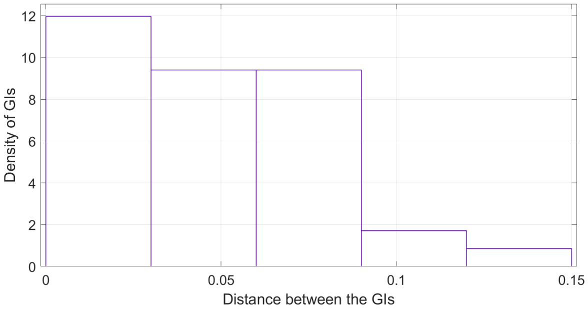

(d) Bordetella pertussis

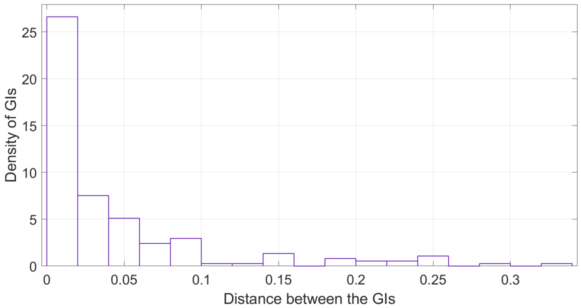

(d) Pseudomonas aeruginosa

Figure S(6) –The distance between the GIs in the most frequent species in the data set (Linear genomes)

Finally, Figure S (7) shows the location distribution of GIs in the linear genomes of the top species in the data set. The difference in the number of the GIs in *Salmonella enterica* compared to other species is considerable. Therefore, we have presented two charts; one with *Salmonella enterica* Figure S(7); and the other one without *Salmonella enterica* Figure S(8) for clarity. In

Figure S(7), in the *Salmonella enterica* distribution, the result is almost the same as in Figure S(5)-b. The elevation of the GIs is more apparent in the terminus, oriC, as well as in the region between 0.4 and 0.6, with the difference between the GIs being just between 30 to 40 GIs. For the other species, as shown in Figure S(8), in*Escherichia coli*, the elevation of the GIs is evident in all regions except the region between 0.1 and 0.3 as well as in the region between 0.9 and 1. Regarding *Klebsiella pneumoniae* and *Pseudomonas aeruginosa*, they have almost the same distribution. The highest density of the GIs in the genome is in the region between 0.3 and 0.4, as well as in the region between 0.7 and 0.8. In the *Bordetella pertussis* GIs chart, the distribution of the GIs is almost uniform excluding a little drop in the region between 0.8 and 0.9. In conclusion, it is difficult to conclude with a few GIs the nature of the locations of GIs in linear genomes of the top species. This is due to the small number of genomes, which in turn, led to obtaining a small number of GIs. Therefore, stating a conclusion here may not reflect the actual picture of the locations of GIs in linear genomes of the top species.

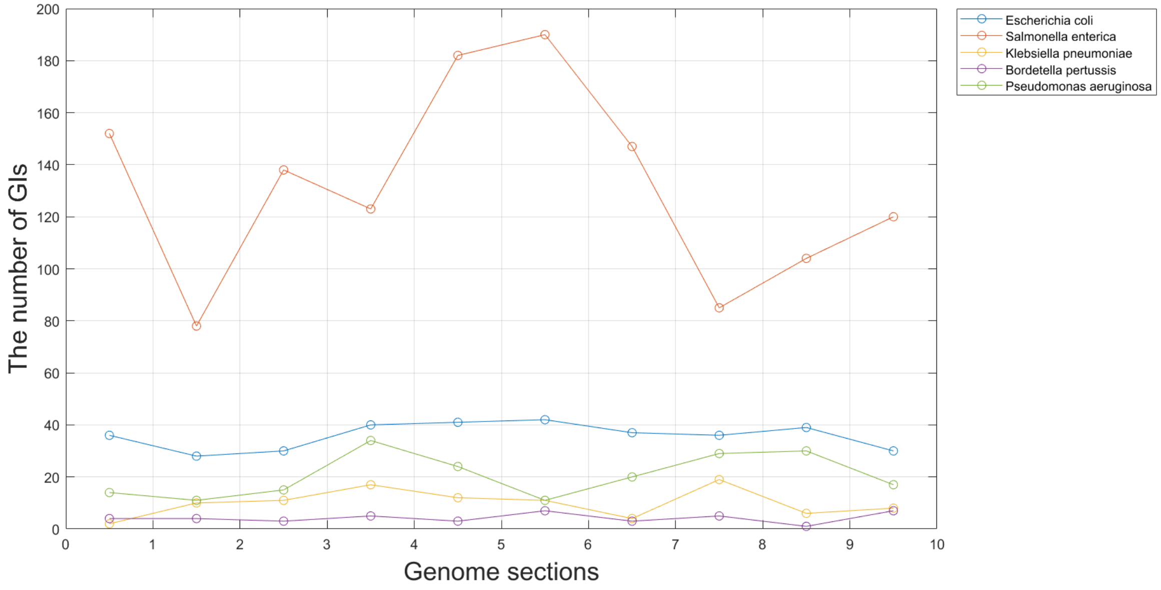

Figure S(7) –The distribution of GIs using the most frequent species in the data set (Linear genomes)

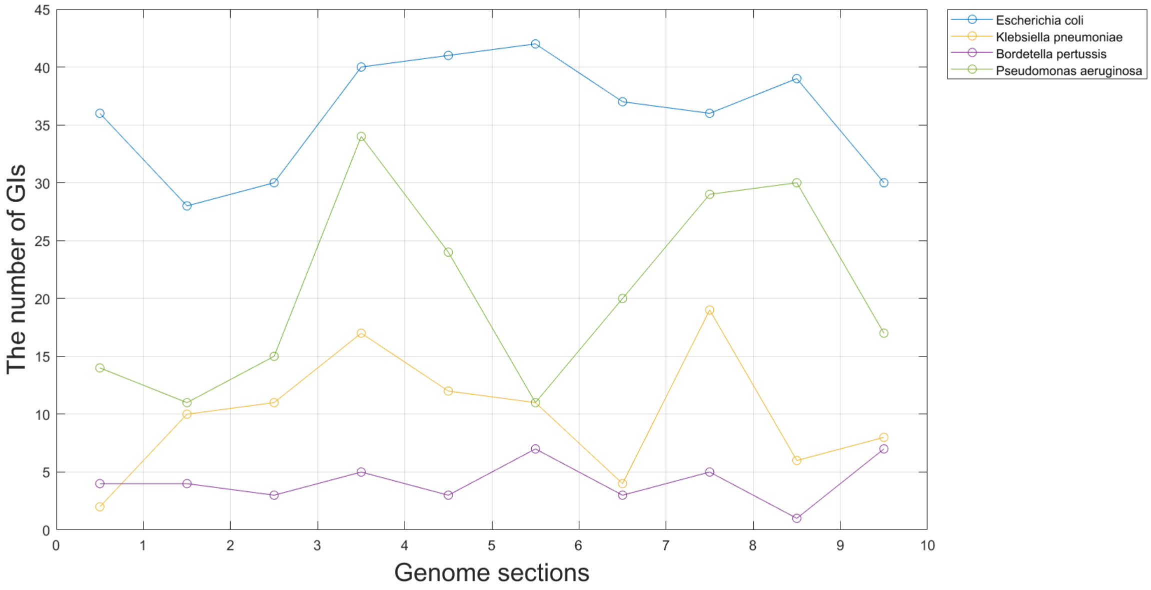

Figure S(8) –The distribution of GIs using the most frequent species in the data set without Salmonella(Linear genomes)
