# Supplementary figures and images for: Investigating the nature of prokaryotic genomic island locations within a genome
Source: PLoS One. 2024 May 2;19(5):e0301172. doi: 10.1371/journal.pone.0301172 (PMC11065298; doi:10.1371/journal.pone.0301172)

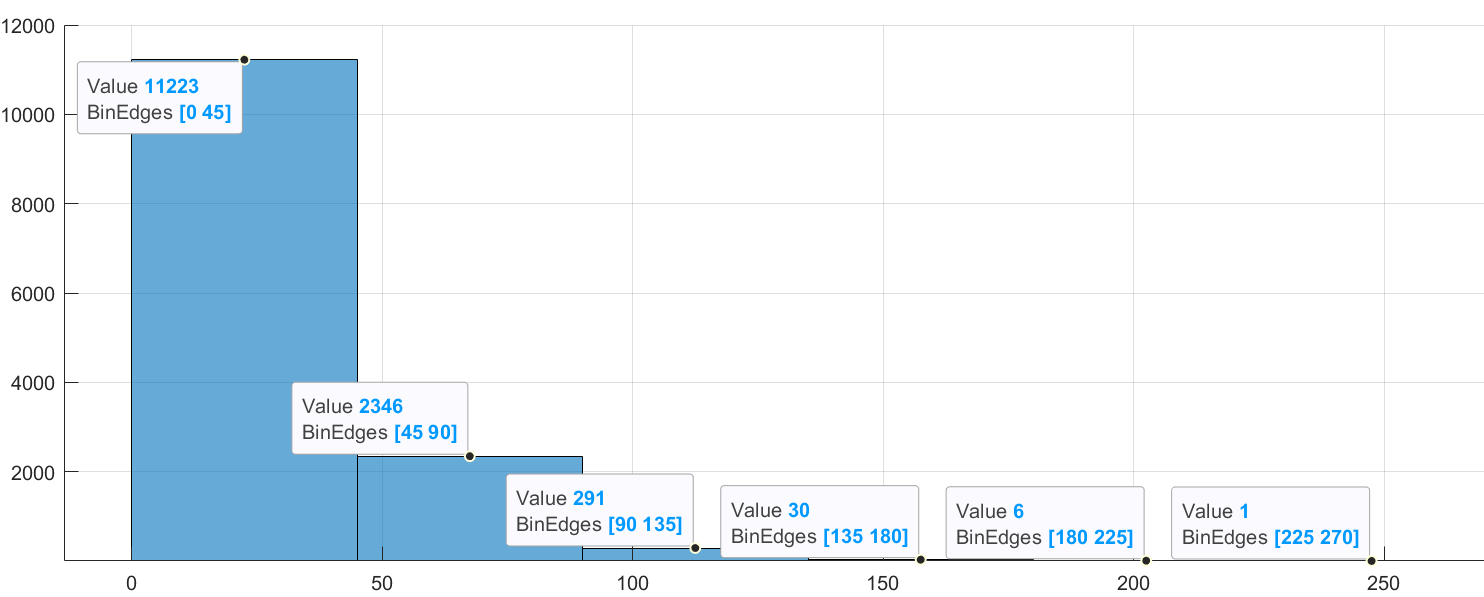

Supplement: S1 Fig — (TIF) [file pone.0301172.s002.tif]

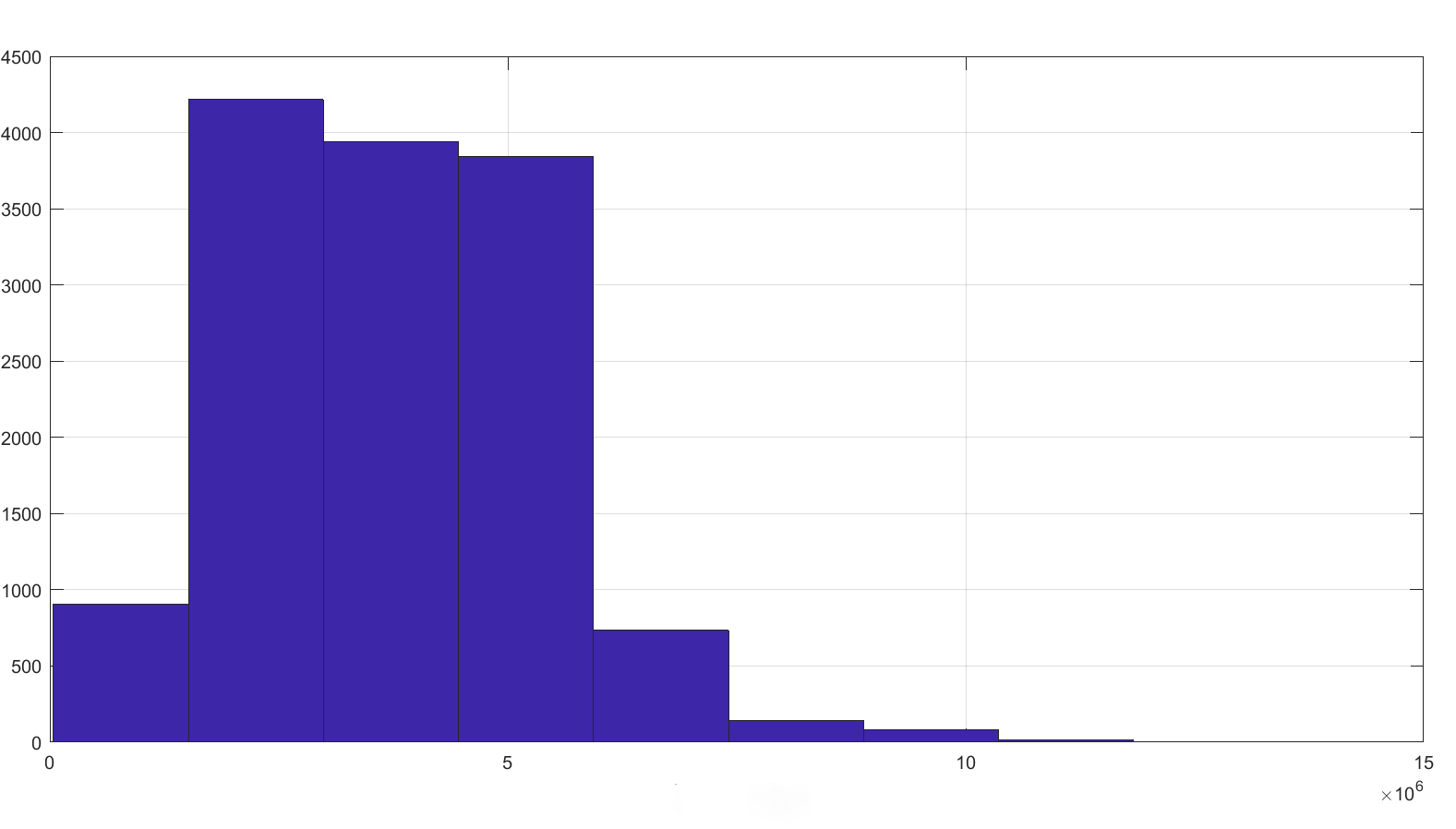

Supplement: S2 Fig — (TIF) [file pone.0301172.s003.tif]

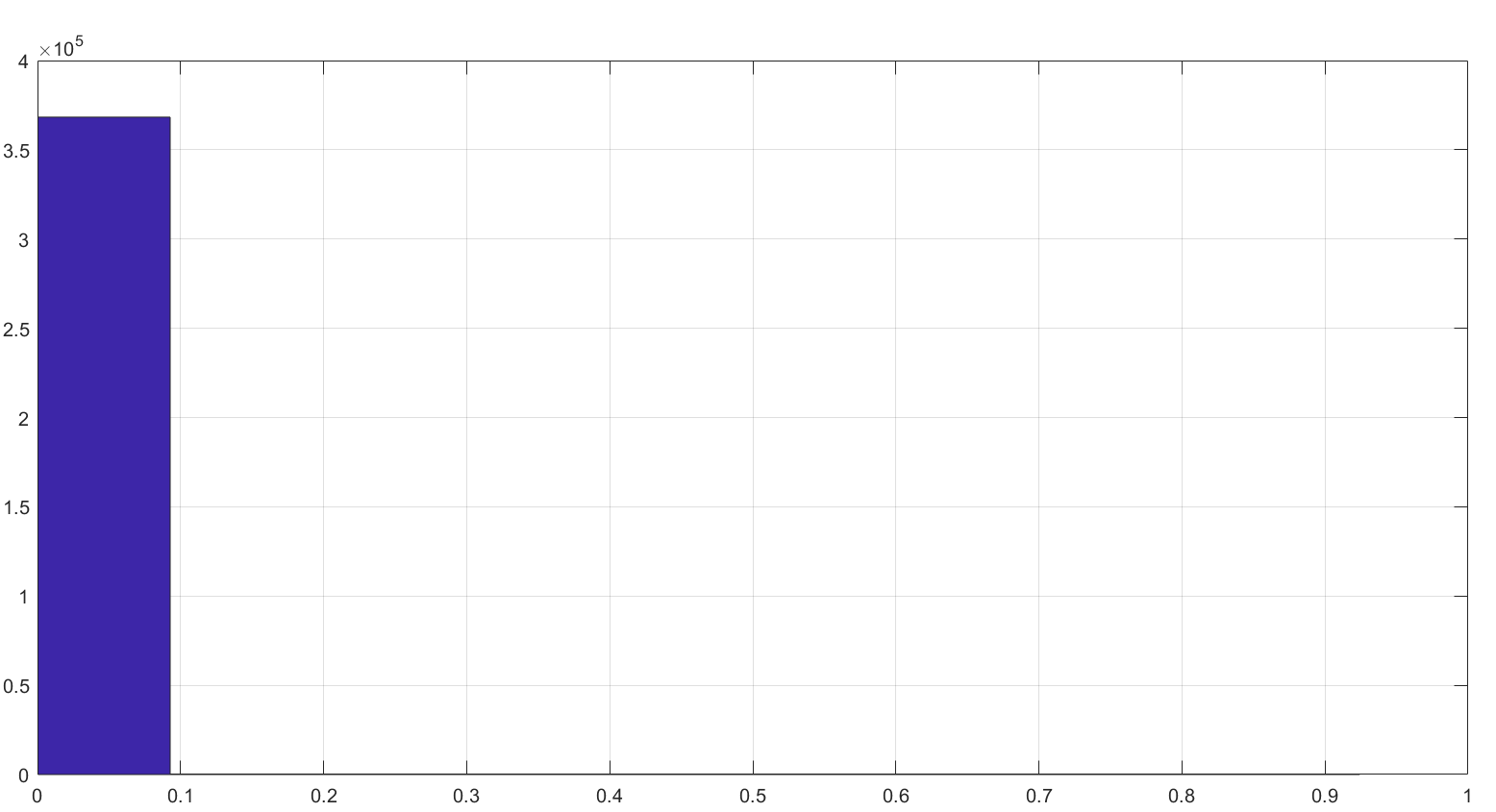

Supplement: S3 Fig — (TIF) [file pone.0301172.s004.tif]

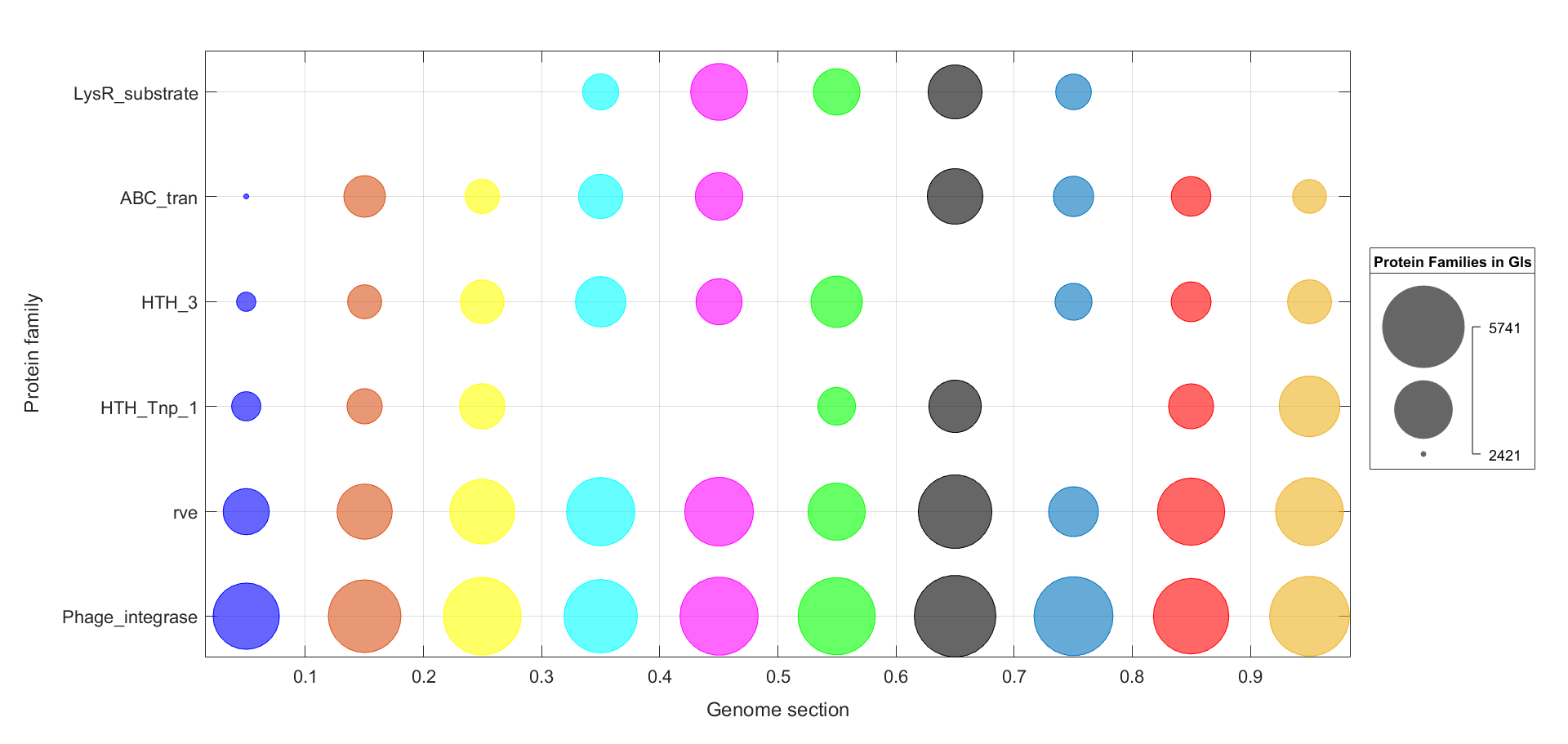

Supplement: S4 Fig — (TIF) [file pone.0301172.s005.tif]

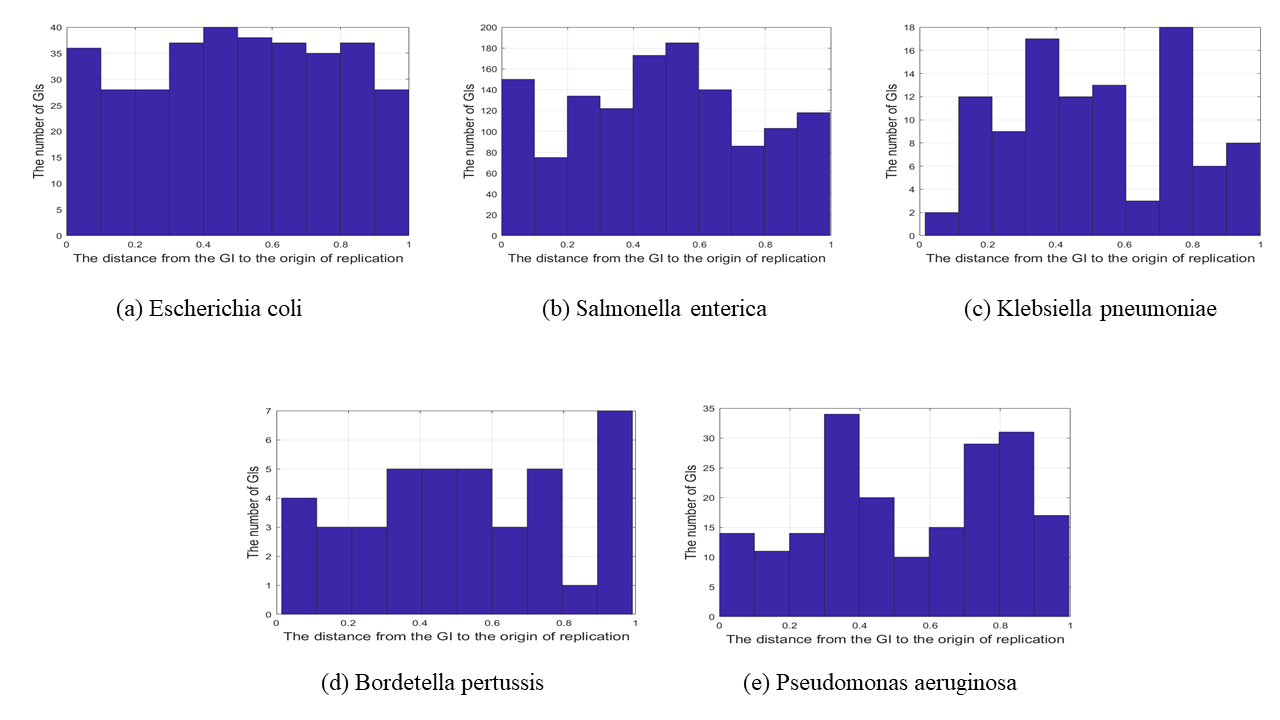

Supplement: S5 Fig — (TIF) [file pone.0301172.s006.tif]

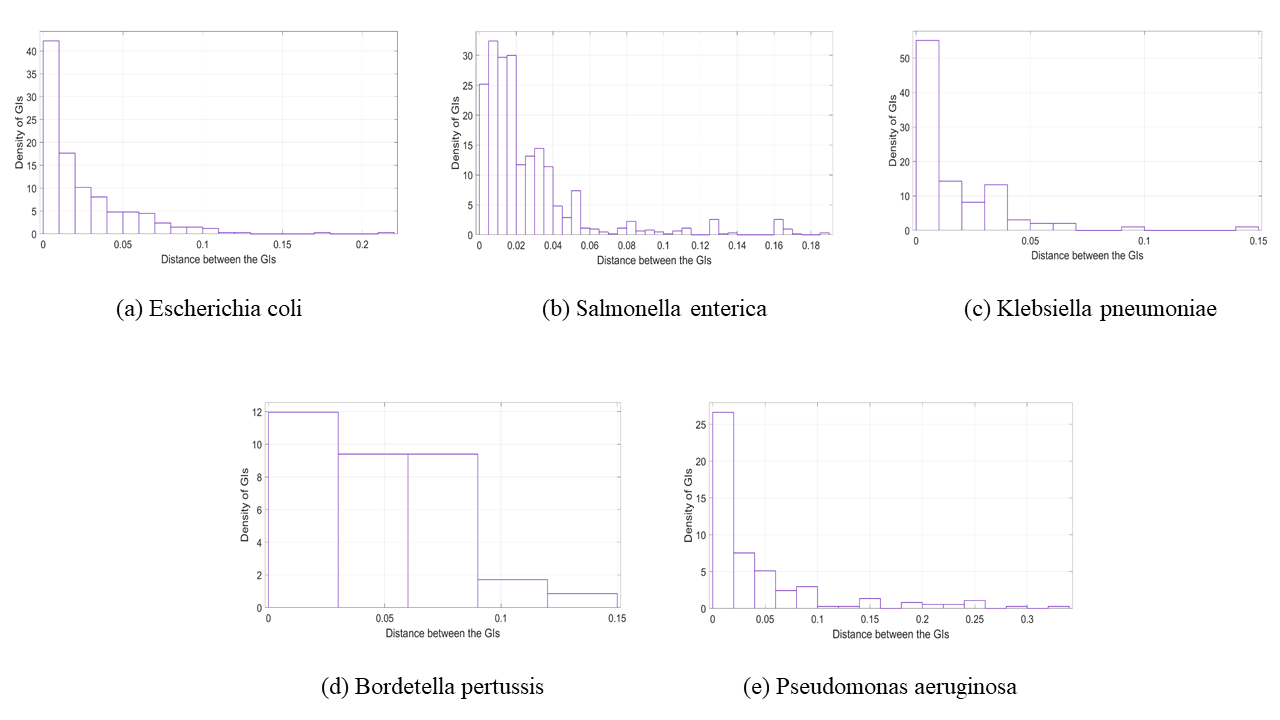

Supplement: S6 Fig — (TIF) [file pone.0301172.s007.tif]

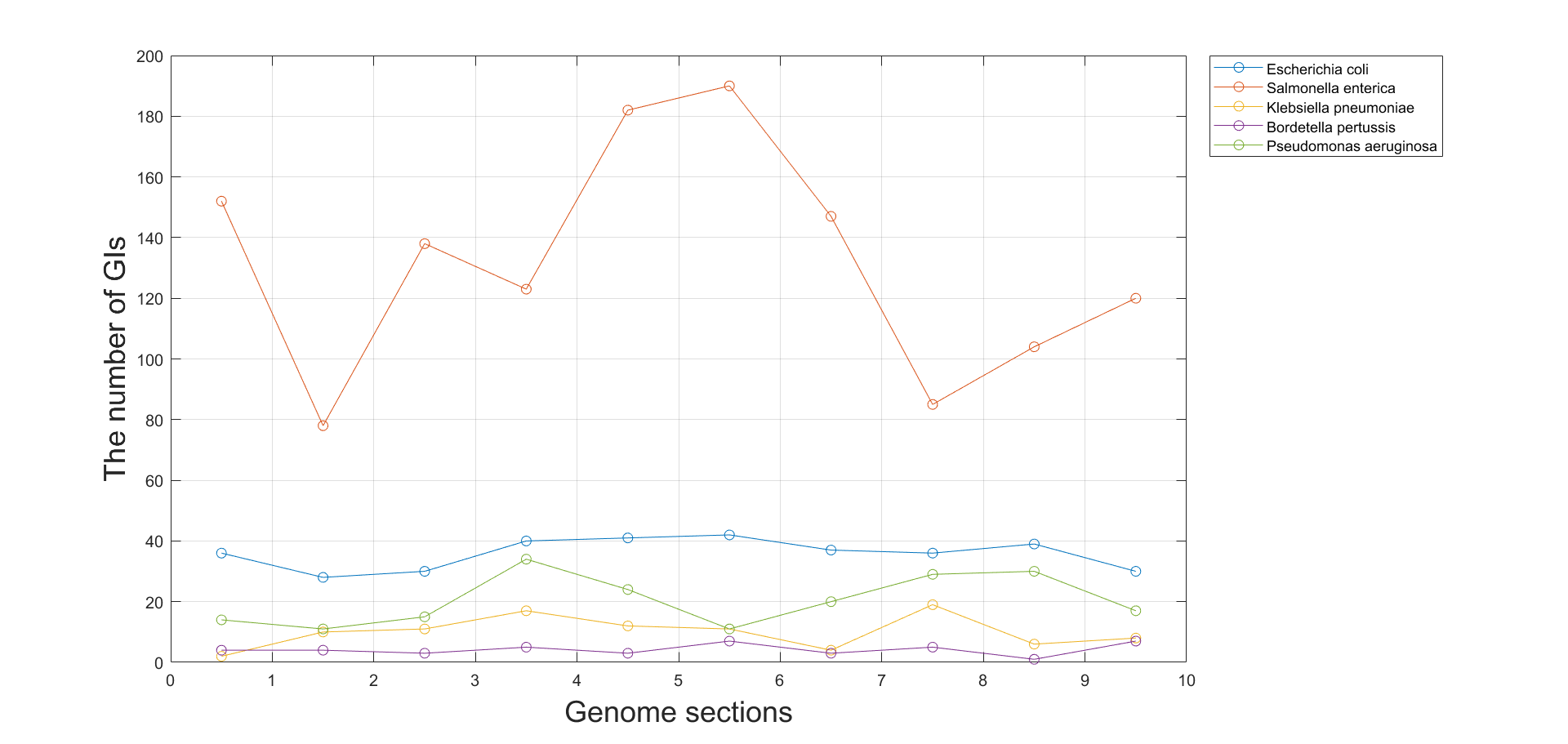

Supplement: S7 Fig — (TIF) [file pone.0301172.s008.tif]

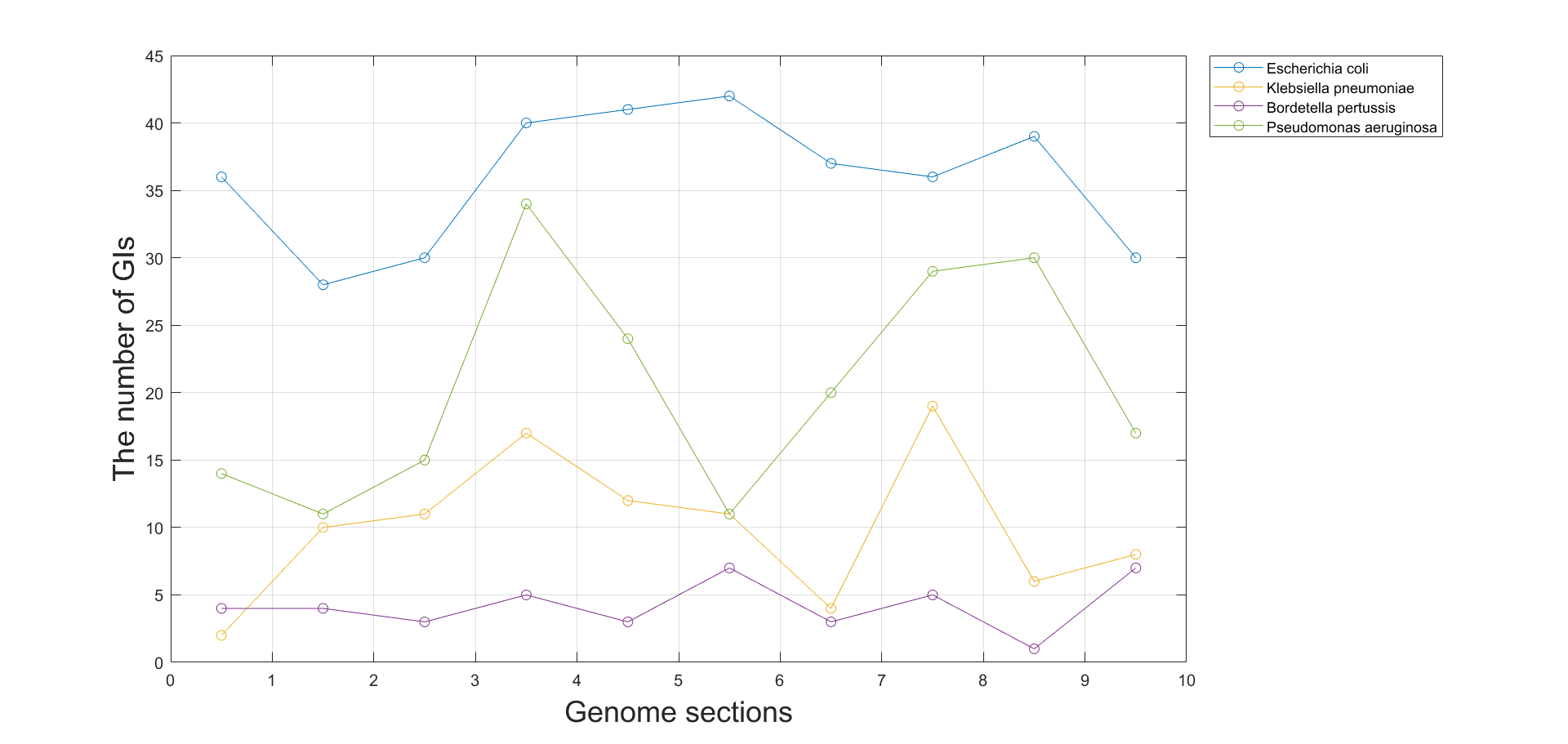

Supplement: S8 Fig — (TIF) [file pone.0301172.s009.tif]
